# Polymer Flooding for EOR: Field Applications, Recovery Performance, Injectivity, and Scale-Up Lessons (2005–2025)

**DOI:** 10.3390/polym18192443

**Published:** 2026-10-07

**Authors:** Rafael E. Hincapie

**Affiliations:** OMV Exploration and Production GmbH, Trabrennstraße 6–8, 1020 Vienna, Austria; rafaeleduardo.hincapiereina@omv.com

**Keywords:** polymer flooding, chemical enhanced oil recovery (EOR), injectivity, polymer retention, field applications, scale-up

## Abstract

Polymer flooding is one of the most widely applied chemical enhanced oil recovery (EOR) technologies and has progressed from conventional onshore sandstone applications to increasingly challenging reservoir environments. This study reviews global polymer flooding experience from 2005 to 2025 using a curated database of 54 documented field applications, including injectivity tests, pilots, and commercial projects across sandstone, carbonate, unconsolidated, heavy oil, offshore, high-salinity, and high-temperature reservoirs. Field data, laboratory investigations, and reported operational experience were evaluated to assess injectivity behavior, polymer retention, viscosity stability, recovery performance, surveillance practices, and implementation outcomes. The reviewed projects span reservoir temperatures from 16 to 124 °C and salinities from approximately 500 to 270,000 ppm total dissolved solids. The analysis shows that successful polymer flood applications are consistently associated with effective mobility control, sustained injectivity, appropriate polymer quality, surveillance-supported reservoir management, and disciplined operational execution. Advances in ATBS-containing and thermally stable polymers have expanded applicability into harsher reservoir conditions, although many developments remain at the injectivity assessment or pilot stage. The resulting synthesis provides a field-based framework linking reservoir conditions, injectivity, polymer performance, surveillance, and operational execution to support screening, qualification, design, scale-up, and decision-making for future polymer flood developments.

## 1. Introduction

Polymer flooding has evolved over the past two decades from a regionally applied enhanced oil recovery (EOR) technique into a globally established mobility control process integrated within modern waterflood management strategies. Once considered primarily a late-stage tertiary recovery option, polymer flooding is now increasingly deployed earlier in field life to improve sweep efficiency, stabilize production, and reduce water handling burdens [1,2]. Numerous field applications across sandstone, unconsolidated, and carbonate reservoirs demonstrate that polymer flooding can provide substantial incremental oil recovery, commonly reported in the range of approximately 8–20% OOIP, while also improving sweep efficiency and production sustainability [3,4,5]. However, field performance varies considerably among projects, reflecting the combined influence of reservoir conditions, injectivity behavior, polymer selection, deployment timing, surveillance practices, and operational execution. Experience from mature developments suggests that polymer flood success depends on the interaction of these factors rather than on any single controlling variable [3,6,7].

Several review papers have documented polymer flooding fundamentals, laboratory behavior, polymer chemistry, and selected field applications [3,8,9,10,11,12]. Nevertheless, most previous reviews focus on specific polymer systems, regional developments, or individual technical aspects, with limited integration of long-term field performance across diverse reservoir settings. In particular, recovery performance, injectivity behavior, polymer retention, operational challenges, surveillance practices, and economic outcomes are rarely evaluated within a single field-oriented framework. Furthermore, publicly available literature is inherently biased toward successful developments, while unsuccessful, discontinued, or inconclusive projects are less frequently reported, making it difficult to establish representative performance envelopes and practical implementation guidance [4,9].

To address these limitations, this paper synthesizes global polymer flooding experience reported between 2005 and 2025 using a curated database of 54 documented applications, including injectivity tests, pilot projects, and commercial developments. The evaluated projects span a wide range of reservoir conditions and provide field-based evidence related to production response, injectivity performance, polymer retention, viscosity stability, mobility control, surveillance outcomes, and economic performance. Rather than focusing on individual success stories or specific polymer chemistries, the analysis emphasizes field performance trends, operational boundaries, recurring challenges, and the factors that influence successful scale-up and long-term deployment.

The objective of this review is to identify field-supported performance envelopes, success factors, failure mechanisms, and implementation lessons relevant to polymer flooding under both conventional and challenging reservoir conditions. Particular emphasis is placed on understanding how injectivity, reservoir severity, operational execution, and project maturity influence recovery performance and commercial outcomes. Reservoir severity is defined here as the combined influence of temperature, salinity, hardness, permeability, oil viscosity, heterogeneity, and injectivity constraints on polymer flood design and execution. The resulting synthesis integrates published field evidence into a practical, field-oriented perspective intended to support screening, design, implementation, and decision-making for future polymer flood developments.

This review contributes beyond traditional polymer flooding summaries by:

- compiling and classifying 54 polymer flood applications (2005–2025);
- differentiating injectivity tests, pilots, and commercial projects;
- benchmarking performance across a wide range of reservoir conditions;
- synthesizing key operational challenges, surveillance practices, and failure modes;
- identifying factors governing scale-up and commercial deployment; and
- establishing an evidence-based framework for project success evaluation.

## 2. Technical Foundations

### 2.1. Physics of Polymer Flooding

Polymer flooding is fundamentally a mobility control process in which polymer addition increases aqueous-phase viscosity and reduces water mobility, thereby improving the mobility ratio between the injected water and displaced oil. Improved mobility control suppresses viscous fingering, delays water breakthrough, enhances areal and vertical sweep efficiency, and promotes more uniform reservoir displacement. Although the endpoint mobility ratio is an important design parameter, favorable field performance may also be influenced by shock front mobility, relative permeability behavior, reservoir heterogeneity, horizontal well geometry, and viscous crossflow effects [1,13,14].

The dominant recovery mechanism depends partly on deployment timing. In secondary-mode applications, polymer flooding primarily improves mobility control and sweep efficiency before extensive channel development occurs. In tertiary-mode applications, polymer is injected after waterflooding has established preferential flow paths, allowing improved mobility control to redistribute flow toward previously unswept oil-bearing zones. Across documented field applications, improved mobility control and sweep efficiency remain the most widely accepted explanations for incremental recovery, although additional mechanisms including viscoelastic effects, elastic instabilities, wettability alteration, low-salinity interactions, and residual oil saturation reduction have also been proposed and continue to be investigated [1,13,14,15,16,17,18,19]. Heavy oil projects such as Pelican Lake have demonstrated that significant recovery improvements can be achieved even when mobility ratios remain greater than unity, provided that polymer injection substantially reduces water mobility and improves sweep efficiency [4,14]. Experimental and numerical studies further indicate that viscous fingering decreases as total mobility is reduced, while fractional flow analyses show that polymer injection retards aqueous front propagation and increases reservoir contact efficiency [15,16].

Deployment timing remains one of the most important design variables in polymer flooding. Laboratory studies, simulation results, and field experience generally indicate that earlier implementation can maximize the benefits of mobility control by preventing the development of severe channeling, excessive water production, and bypassed oil accumulation [1,5,14]. Secondary polymer flooding, implemented before extensive waterflood maturity, often requires lower cumulative polymer consumption to achieve a given sweep improvement because mobility control is established before dominant flow paths become entrenched. However, field evidence also demonstrates that polymer flooding can remain effective when implemented later in reservoir life, particularly where significant mobile oil remains and opportunities exist to improve sweep efficiency through flow redistribution. Consequently, no universal optimum timing can be defined across all reservoirs. Instead, timing decisions should be guided by reservoir-specific factors such as water cut, remaining oil saturation, mobility ratio, sweep efficiency, injectivity conditions, reservoir heterogeneity, and economic objectives.

Figure 1 illustrates the conceptual relationship between polymer rheology, reservoir position, Darcy velocity, and apparent shear rate during polymer flooding. Polymer solutions generally exhibit near-Newtonian or shear-thinning behavior under most reservoir flow conditions. Although apparent shear-thickening and viscoelastic responses have been observed in laboratory studies at elevated flow velocities [17], published field analyses indicate that most reservoir volumes experience Darcy velocities of approximately 0.01–0.2 ft/day, typically below conditions associated with strong viscoelastic responses [17,18,20]. The velocity ranges shown in Figure 1 represent generalized reservoir-scale conditions and should be interpreted as conceptual rather than universal values. Local velocities can vary significantly depending on well architecture, completion design, reservoir heterogeneity, and near-wellbore flow geometry. In particular, horizontal wells, which are widely used in several modern polymer flooding projects, may exhibit different local velocity distributions compared with conventional vertical wells. While horizontal wells generally reduce flux per unit completion length and improve areal sweep efficiency, localized high-velocity regions may still occur near perforations, fractures, or inflow points. Consequently, the figure is intended to illustrate general trends in flow regime evolution rather than to define fixed velocity boundaries applicable to all well configurations.

Polymer transport is influenced by adsorption, mechanical retention, hydrodynamic dispersion, and inaccessible pore volume (IPV). IPV has historically been included in transport models to explain accelerated polymer breakthrough; however, its significance remains debated and appears highly reservoir-dependent. Recent studies suggest that IPV may be negligible in many higher-permeability reservoirs, where polymer molecules can access most of the pore network [21]. Advances in polymer chemistry have expanded polymer flooding into higher-temperature, higher-salinity, carbonate, offshore, and heavy oil reservoirs by improving thermal stability, salinity tolerance, and mechanical robustness [22,23]. While laboratory studies have reported additional pore-scale recovery mechanisms, including viscoelastic effects and elastic instabilities [19,24,25], field evidence continues to support mobility control, sweep improvement, and conformance enhancement as the primary mechanisms responsible for commercial polymer flood performance. Therefore, viscoelasticity is discussed separately in this review, where laboratory observations and field-scale evidence are critically evaluated.

It is important to distinguish between polymer rheology measured in bulk fluids and apparent rheological behavior observed during flow through porous media. Bulk rheology is typically characterized using rotational or extensional rheometers and describes properties such as shear thinning, viscoelasticity, and shear thickening under controlled laboratory conditions. In porous media, however, the observed pressure response reflects the combined influence of bulk polymer properties, pore geometry, flow convergence and divergence, mechanical retention, inaccessible pore volume, and flow redistribution effects. Consequently, apparent rheological responses inferred from porous media experiments cannot be interpreted solely from rheometer measurements. This distinction is particularly relevant when evaluating reports of shear thickening, viscoelasticity, or mobility control behavior under reservoir conditions.

The velocity ranges illustrated in Figure 1 represent generalized field conditions derived primarily from conventional reservoir-scale flow analysis. However, local velocities may vary significantly depending on well architecture, completion design, and near-wellbore flow geometry. In particular, horizontal wells, which are widely used in several modern polymer flooding projects including heavy oil developments, may exhibit different local velocity distributions compared with conventional vertical wells. While horizontal wells can reduce flux per unit completion length and improve areal sweep efficiency, localized high-velocity regions may still occur near perforations, fractures, or completion inflow points. Consequently, the velocity ranges shown in Figure 1 should be interpreted as representative conceptual values rather than universal limits applicable to all well configurations.

### 2.2. Polymer Types and Advances Enabling Expanded Reservoir Applicability

HPAM remains the dominant polymer used in chemical EOR because of its high viscosifying efficiency and favorable economics, although its performance can be limited by thermal hydrolysis, divalent ion sensitivity, and mechanical degradation [1,8]. Advances in polymer manufacturing and molecular weight control have improved injectivity and stability compared with earlier formulations. To extend application into harsher environments, ATBS-based, sulfonated, and NVP-containing polymers have been developed. Sulfonated monomers improve thermal stability, maintain viscosity in hard brines, and reduce precipitation risks under elevated temperatures and salinities [1,26]. Laboratory studies have demonstrated viscosity retention at temperatures approaching 95–120 °C and salinities exceeding 100,000 ppm, supporting their application in high-temperature/high-salinity (HTHS) reservoirs [23,27]. Industry developments and independent assessments identify ATBS/NVP copolymers and selected associative polymers among the leading candidates for HTHS applications [28]. These advances have expanded the technical feasibility of polymer flooding, although many HSHT developments remain at the injectivity test or pilot stage, with fewer commercially mature recovery projects reported.

Associative polymers contain hydrophobic groups that create reversible intermolecular networks, providing enhanced viscosity and elasticity at relatively low concentrations. While these characteristics may improve mobility control, associative polymers can also generate higher resistance factors and retention, requiring careful injectivity management and implementation strategies [1,20,29,30,31].

Biopolymers have also been investigated as alternatives to synthetic polyacrylamides because of their inherent salinity tolerance and environmental compatibility. Xanthan gum remains the most widely studied biopolymer for EOR applications and exhibits favorable viscosity retention under high-salinity conditions. However, broader field deployment has been constrained by microbial degradation, injectivity limitations associated with larger molecular structures, cost considerations, and a more limited field performance database compared with HPAM-based systems. Consequently, synthetic HPAM and ATBS-modified polymers continue to dominate commercial polymer flood developments, while biopolymers remain primarily application-specific alternatives or niche candidates under selected reservoir conditions [8,13].

Over the last two decades, polymer development has progressed less through the introduction of entirely new polymer families and more through incremental improvements in molecular architecture, thermal stability, salinity tolerance, dissolution behavior, and injectivity performance. The resulting evolution has expanded polymer flooding from conventional moderate-temperature sandstone reservoirs toward offshore, heavy oil, carbonate, and high-salinity/high-temperature environments, while HPAM and its modified derivatives remain the dominant commercial polymer platform.

Emerging systems, including polymer–surfactant formulations, polymer–nanoparticle systems, and thermoresponsive polymers, seek to combine mobility control with additional mechanisms such as interfacial tension reduction, wettability alteration, or improved mechanical robustness. Recent studies have reported synergistic interactions between amphoteric surfactants and ATBS-based polymers, although field experience remains limited [32]. Importantly, successful polymer injection should not be equated with commercial success, which additionally requires sustained recovery improvement, operational reliability, and favorable project economics.

Polymer product form is an additional selection variable. Commercial polymers are supplied as dry powders or liquid formulations, including inverse emulsions. Dry powders generally offer higher active polymer content, whereas liquid products can simplify handling and reduce surface facility requirements. Consequently, polymer selection depends not only on chemistry but also on project scale, logistics, facility constraints, dissolution quality, injectivity performance, and economics [33,34]. Case studies demonstrate that powder and liquid systems may exhibit different injectivity and operating characteristics under specific reservoir conditions, emphasizing the need for integrated technical and economic qualification. Recent modular preparation and injection systems have further reduced many of the logistical constraints historically associated with dry polymer deployment [34,35,36].

### 2.3. Reservoir Conditions and Polymer Qualification Requirements

Reservoir temperature, salinity, hardness, rock mineralogy, and permeability define the operating environment to which polymer systems must be qualified and adapted. Consequently, successful polymer flooding depends not only on reservoir conditions but also on selecting polymer chemistry, molecular weight, concentration, and water treatment strategies that are compatible with those conditions. Temperature accelerates HPAM hydrolysis, reducing viscosity and increasing sensitivity to divalent ions [15]. Screening guidelines generally recommend HPAM below ~75 °C, ATBS-rich polymers between 75 and 95 °C, and NVP- or high-ATBS formulations above 100 °C [1]. Laboratory studies and Arrhenius-based analyses indicate that some high-ATBS polymers may retain useful viscosity for more than five years at 120 °C under oxygen-free hard-brine conditions [21]. Salinity and hardness affect polymer coil expansion, viscosity, and precipitation risk, while sulfonated polymers exhibit improved tolerance to harsh brines [8,22]. AMPS/NVP-modified polymers have demonstrated stability at TDS levels exceeding 150,000–200,000 ppm, and modified water/polymer studies highlight the importance of ion composition, including sulfate, for polymer rheology and recovery performance [23,37].

Polymer retention results from adsorption, mechanical entrapment, and, in some cases, inaccessible pore volume (IPV). Adsorption is generally irreversible and increases in clay-rich formations, particularly those containing smectite [38]. Recent studies suggest that IPV may be less significant in many commercial floods developed in reservoirs with permeabilities above approximately 300 mD, although its magnitude remains dependent on polymer properties, rock type, salinity, flow conditions, and interpretation methodology [21]. Retention assessment in carbonates, especially under high-temperature/high-salinity conditions, requires careful analytical procedures, as demonstrated by recent ATBS polymer studies that corrected viscosity-based measurements for degradation effects [39].

Thermal, mechanical, oxidative, and biological degradation can compromise polymer integrity and must be controlled throughout field operations. Mechanical degradation is most commonly associated with high-shear surface equipment such as pumps, valves, chokes, and mixing systems, although additional degradation may occur in wellbores, perforations, and near-wellbore regions where flow convergence and elevated local shear conditions are present. Oxidative degradation is accelerated by oxygen and transition metals [1]. Detailed rheological investigations further show that shear- and extension-dominated flows produce different degradation pathways and pressure responses, emphasizing the importance of reservoir-relevant polymer qualification [40]. Together, these factors govern polymer stability, propagation, injectivity, and recovery performance. Figure 2 summarizes the evolution of temperature, fluid velocity, and polymer viscosity along the injection-to-reservoir flow path.

### 2.4. Viscoelasticity: Laboratory Evidence Versus Field Evidence

Polymer viscoelasticity has been proposed as a mechanism that may improve microscopic displacement efficiency beyond conventional mobility control. Laboratory studies have reported reductions in residual oil saturation (Sor), enhanced oil mobilization, and pore-scale displacement phenomena associated with elastic stresses generated as polymer molecules deform while flowing through pore constrictions [17,24,41,42,43,44]. These observations have stimulated extensive research into extensional rheology, elastic instabilities, and pore-scale recovery mechanisms; however, direct field-scale validation remains limited. Recent evaluations of commercial polymer floods indicate that reservoir velocities and pressure gradients are generally below the onset conditions associated with strong viscoelastic responses observed in laboratory experiments. Azad and Seright [18] showed that field Darcy velocities are typically much lower than those required for significant viscoelastic Sor reduction and that only a small fraction of reservoir volume is likely to experience such conditions. Consequently, laboratory observations of elastic oil mobilization should not be assumed to translate directly into reservoir-scale recovery.

This does not invalidate laboratory evidence but highlights the importance of scale-dependent interpretation. Incremental recovery during polymer flooding may result from multiple interacting mechanisms, including improved mobility control, sweep efficiency, flow redistribution, wettability alteration, low-salinity effects, and reservoir heterogeneity [45,46,47,48]. Although reductions in residual oil saturation have been reported under controlled laboratory conditions, most documented field performance can be adequately explained through established mechanisms such as mobility control, sweep improvement, and conformance effects. Therefore, viscoelasticity should be regarded as a laboratory-supported pore-scale mechanism whose quantitative contribution to reservoir-scale recovery remains uncertain, while field evidence continues to provide stronger support for mobility control and sweep efficiency improvements as the primary drivers of polymer flood performance [18].

## 3. Data Sources, Methodology, and Project Classification

### 3.1. Data Sources and Scope

An overview of the dataset composition is provided in Table 1. The curated database includes 54 polymer flooding applications from 20 countries, comprising injectivity tests, pilots, commercial developments, and supporting studies compiled from conference papers, journal publications, and publicly available field reports. Mature commercial projects such as Daqing, Pelican Lake, Mangala, Marmul, Captain, and Kalamkas were evaluated alongside pilot and injectivity-focused applications to capture both long-term deployment experience and operational learning. While commercial projects provide the strongest evidence of technology maturity, pilot and injectivity trials remain valuable for identifying injectivity constraints, polymer stability requirements, and scale-up risks.

Only projects containing sufficient publicly available technical information for comparative evaluation were retained. Reported values, ranges, and qualitative observations were preserved as published, and missing parameters were not estimated. Projects were classified according to reservoir type, development stage, polymer chemistry, operating environment, and thermo-chemical severity. Extracted variables included reservoir properties, polymer type, injectivity behavior, retention, viscosity stability, recovery performance, economic information, and reported operational challenges.

To reduce scale-related bias, pilot and commercial projects were evaluated separately, while supporting studies and projects with unclear development status were retained as independent categories. Comparative analyses focus on operating envelopes, implementation outcomes, and recurring technical trends rather than ranking individual projects. Consequently, the database should be interpreted as a literature-based benchmarking dataset rather than a complete global census of polymer flooding projects. Detailed project information is provided in Appendix A.1, Appendix A.2 and Appendix A.3.

### 3.2. Database Construction, Benchmarking Approach, and Limitations

The database was compiled from publicly available polymer flooding literature published between 2005 and 2025, including SPE papers, peer-reviewed journals, industry reports, and documented field case histories. Projects were identified through structured searches of SPE OnePetro, Scopus, Web of Science, and Google Scholar using keywords related to polymer flooding, polymer EOR, injectivity, retention, degradation, surveillance, heavy oil, offshore, carbonate, and high-salinity/high-temperature applications.

Project selection followed a two-stage screening process. First, documented injectivity tests, pilot projects, and commercial polymer flood developments were identified. Second, projects were retained only when sufficient technical information was available to support comparative evaluation of reservoir conditions, polymer performance, injectivity, operational experience, project maturity, or recovery outcomes. Projects with insufficient disclosure were excluded. The final database comprises 54 documented applications spanning injectivity tests, pilots, and commercial developments. Projects were classified by maturity because project objectives differ substantially. Injectivity tests primarily evaluate polymer injectivity and propagation, pilots assess limited-scale field performance, and commercial projects provide evidence of long-term injectivity sustainability, operational reliability, surveillance effectiveness, scale-up feasibility, produced water management, and economic performance. Consequently, injectivity-focused projects were not used as recovery performance benchmarks. Normalization was limited to common engineering descriptors, including temperature, salinity, permeability, oil viscosity, polymer type, and implementation stage. Missing values were not estimated or interpolated, and recovery metrics were retained as originally reported because definitions varied among studies. Therefore, results are presented as field-observed trends and performance ranges rather than direct quantitative rankings.

The database is subject to publication and disclosure bias because successful or well-documented projects are more likely to be reported than unsuccessful or discontinued applications. Reporting completeness also varies considerably among sources. To minimize interpretation bias, author-reported data were retained without recalculation whenever possible. Accordingly, the database should be regarded as an evidence-based benchmarking dataset rather than a statistically representative sample of global polymer flooding activity.

Interpretation of technology maturity relies primarily on evidence from large-scale commercial developments, including Daqing, Pelican Lake, Mangala, Marmul, Captain, and Kalamkas, which provide the most comprehensive information on long-term field performance. Collectively, these projects demonstrate that sustained polymer flood implementation is no longer restricted to a single region but has been successfully deployed across China, Canada, India, Oman, Kazakhstan, and the North Sea under a broad range of reservoir and operating conditions. Unless otherwise stated, observations and trends discussed throughout this review are derived from the field cases included in the database, while the interpretive frameworks and recommendations represent the authors’ synthesis of the published literature and reported field outcomes.

### 3.3. Data Availability Statement

The database supporting this review was compiled from publicly available literature sources listed in the references and summarized in Appendix A.1, Appendix A.2 and Appendix A.3. The dataset supporting the conclusions of this study is available from the corresponding author upon reasonable request.

## 4. Global Evolution of Polymer Flooding (2005–2025)

Field applications for polymer flooding have expanded significantly over the past two decades. The process has evolved from a regional EOR technique into a globally standardized mobility control process [1]. It is increasingly positioned as an optimized waterflooding approach rather than a late-stage tertiary recovery option [2,49]. Early deployments were dominated by onshore sandstone reservoirs under moderate temperature and salinity conditions. China established the operational benchmark through sustained full-field polymer flooding at Daqing and related assets, representing the largest and most extensively documented polymer flood developments worldwide [5,49,50,51]. Long-term field experience from Daqing, Pelican Lake, Mangala, Marmul, and other commercial developments demonstrates that polymer flooding can be implemented and sustained over periods exceeding a decade while maintaining injectivity, mobility control, and operational performance. Several of these developments progressed from pilot testing to field-wide implementation involving hundreds of wells, large injected polymer volumes, and extensive surveillance programs, providing some of the strongest publicly documented evidence of polymer flood maturity [4,5,52]. These field results, together with decades of commercial deployment across multiple regions, support the classification of polymer flooding as a mature and proven mobility control technology rather than an experimental recovery process. However, recent analyses emphasize that the most successful outcomes are concentrated in a limited number of large-scale projects and favorable reservoir settings and therefore should not be regarded as representative of all chemical EOR applications [53]. Consequently, field performance remains strongly dependent on reservoir quality, injectivity, operational discipline, and project execution.

Over the last decade, the global polymer flooding portfolio has diversified as waterflood efficiency has declined in mature assets [11]. Improved polymer chemistries and pressure to reduce water handling have further accelerated adoption. The dataset analyzed shows expansion into heavy oil reservoirs, extensively documented by Delamaide [4], offshore environments, and high-salinity/high-temperature systems (e.g., [54,55]). Despite this expansion, successful projects remain strongly biased toward sandstone lithologies [2]. This evolution reflects a shift toward sweep improvement, production stabilization, and lifecycle value optimization. Polymer flooding is increasingly favored where thermal or surfactant-based EOR methods are impractical.

As shown in Figure 3, documented projects now span a substantially wider operating envelope than early polymer flood applications, including heavy oil, offshore, carbonate, and HSHT reservoirs. The implications of this evolution beyond traditional screening criteria are discussed later in the paper. The figure further highlights the progression from conventional sandstone developments to offshore and carbonate reservoirs, enabled by advances in polymer chemistry and injectivity management. Despite this expansion, full-field commercial implementation remains concentrated within a limited number of sandstone projects, whereas most carbonate and high-temperature/high-salinity applications remain at the pilot or technology qualification stage. This trend illustrates both the increasing technical maturity of polymer flooding and the continuing challenges associated with extending the technology to more severe reservoir conditions. Although many documented applications remain at the pilot or qualification stage, several projects have progressed to long-term commercial operation. Their maturity is reflected by sustained injection histories, large treated reservoir volumes, and documented surveillance-supported production responses.

### 4.1. Geographic and Reservoir Trends

Long-term commercial-scale polymer flooding has been concentrated in a limited number of regions, particularly China, North America, Europe, and selected Middle Eastern developments, although additional pilots and field applications have also been reported in Latin America and other regions [5]. Aladasani et al. [53] identified Daqing as one of the few large-scale, highly homogeneous reservoirs where polymer flooding has delivered relatively consistent recovery gains. The same study emphasizes that such outcomes represent exceptional cases and should not be generalized to the broader global polymer flooding population. Outside China, adoption is fragmented but increasing, with projects typically implemented after extended waterflooding maturity and relying on pilots to derisk injectivity and polymer stability [11]. Kuwait, for instance, has several different pilots (long-term injectivity trials) using acrylamido–tertiary–butyl–sulfonate (ATBS)-based polymers [56,57]. Latin America has also contributed several documented polymer flooding applications, including projects in Argentina, Brazil, Colombia, and Venezuela, demonstrating the applicability of polymer flooding across diverse reservoir settings and development stages.

Sandstone reservoirs dominate successful polymer floods in both number and performance consistency [2]. Field and laboratory evidence shows lower retention uncertainty and more predictable polymer propagation above approximately 500 mD permeability. Heavy oil reservoirs constitute an important growth segment, although the associated technical and operational considerations are discussed later in the paper. These challenges intensify under high-salinity and high-temperature conditions, where technology-driven and tailor-made approaches have been used in some projects (e.g., [55,58]).

### 4.2. Emerging Growth Regions: Latin America and India

The reviewed database demonstrates that documented polymer flood activity now spans North America, South America, Europe, the Middle East, Central Asia, Southeast Asia, and offshore developments, illustrating a substantially broader geographic footprint than that represented in earlier reviews focused primarily on China and North America. This has been one of the most significant developments during the last decade. In Latin America, polymer flooding activity has expanded rapidly across a range of reservoir settings. Large-scale projects and pilots have been reported in Suriname, Colombia, and Argentina. Colombian applications include Yariguí-Cantagallo, Casabe, and Chichimene, while Argentina has reported multiple large-scale developments, including Diadema, El Corcobo, Desfiladero Bayo, and El Trébol. These projects collectively demonstrate the adaptability of polymer flooding under diverse reservoir conditions and operating environments [14].

India has likewise become an important deployment area. Large-scale polymer flooding programs have been implemented in Mangala, Bhagyam, Aishwariya, Bechraji, and Mehsana, among others. These developments have demonstrated sustained field-scale implementation under challenging operational conditions and have contributed significantly to global polymer injection volumes [14]. The emergence of these regions illustrates an important industry trend: polymer flooding has evolved from a technology adopted primarily in a few specialized areas into a globally deployed mobility control process applicable across a broad range of geological settings. Recent growth has been driven less by new polymer chemistries than by improved project execution, water-management practices, surveillance capabilities, and confidence gained from successful field-scale implementations.

### 4.3. Technology and Strategy Shifts

Technological evolution since 2005 has primarily reflected incremental refinement rather than fundamental changes in polymer flooding mechanisms [1]. Conventional HPAM remains the dominant polymer because it generally provides the most favorable balance between viscosity generation, injectivity, field experience, availability, and cost. Advances in oxygen control, water treatment, molecular weight management, polymer quality assurance, surface handling, hydration quality, and shear management have progressively extended the operational envelope of HPAM-based systems, highlighting that polymer stability often depends as much on operational control as on polymer chemistry itself [59]. ATBS-modified and sulfonated polymers have further expanded applicability in high-temperature and high-salinity reservoirs through improved resistance to hydrolysis, divalent ions, and thermal degradation [21,60]. However, under increasingly severe reservoir conditions, the ATBS content required to maintain long-term stability may become sufficiently high to increase polymer costs and reduce economic competitiveness relative to conventional formulations [21]. NVP-containing polymers have demonstrated improved thermal stability in laboratory studies, but their lower molecular weight and limited field adoption have restricted their role as primary mobility control agents in large-scale floods. Consequently, most recent field developments continue to rely on HPAM or ATBS-modified HPAM systems rather than entirely new polymer families [21,61]. Emerging technologies, including delayed-viscosity and encapsulated polymers, seek to improve injectivity preservation under extreme shear conditions, particularly in offshore and subsea applications where operational constraints increasingly govern field performance [62,63].

Strategically, recent developments have placed greater emphasis on operational reliability, injectivity management, and disciplined project execution. Modern projects seem to prioritize early injectivity and predictable propagation over aggressive viscosity targets to support long-term success. Polymer flooding is increasingly positioned as a low-risk intervention for mature waterfloods with high water-handling burdens. Project value is now justified primarily by water-cut reduction, production acceleration, and emissions intensity improvements [62].

### 4.4. Onshore and Offshore Implementation Considerations

Polymer flooding has been implemented in both onshore and offshore environments, although offshore developments introduce additional constraints related to platform space, chemical storage and handling, logistics, produced water management, and facility footprint. These factors influence polymer selection, preparation systems, supply chain reliability, and water treatment requirements. While the fundamental recovery mechanism remains unchanged, offshore projects generally require greater emphasis on facilities integration, operational simplicity, injection system robustness, and long-term maintainability. Experience from developments such as Dalia and other offshore pilots demonstrates that these challenges can be addressed through fit-for-purpose facility design, modular chemical-handling systems, and robust operational practices (e.g., [3,64,65,66,67]).

### 4.5. Monitoring, Surveillance, and Recovery Attribution

Polymer flood performance is typically evaluated through integrated surveillance programs designed to verify polymer propagation, mobility control effectiveness, sweep efficiency improvement, and incremental oil recovery. Common surveillance indicators include injection pressure trends, injectivity evolution, polymer concentration measurements in produced fluids, water-cut response, oil rate changes, voidage replacement performance, and pattern-level material balance analysis. In many projects, tracer tests, interwell connectivity studies, pressure-transient analysis, and history-matched reservoir simulation are additionally used to assess polymer movement and identify changes in flow distribution within the reservoir.

Importantly, improved recovery is rarely attributed to polymer injection solely on the basis of increased oil production. Instead, operators commonly integrate multiple lines of evidence, including delayed water breakthrough, reduced water–oil ratio, sustained production response, pressure behavior, polymer breakthrough observations, and comparison with established waterflood performance. Mature projects such as Daqing, Captain, Pelican Lake, Marmul, and Milne Point have demonstrated that long-term surveillance is essential for distinguishing polymer-related sweep improvements from normal field management effects and for quantifying incremental recovery with greater confidence. Consequently, polymer flood success is increasingly assessed through continuous surveillance and reservoir management workflows rather than production metrics alone (e.g., [4,9,52,67,68]). Polymer flood implementation typically progresses from reservoir screening and laboratory qualification through injectivity testing, pilot evaluation, staged field expansion, and mature flood optimization. Throughout the project lifecycle, water quality management, polymer chemistry, facility design, surveillance, and economic assessment remain continuous activities influencing project success.

### 4.6. Evolution of Polymer Flooding Beyond Traditional Screening Criteria

The evolution of polymer flooding over the last two decades has been characterized by the reassessment of several screening assumptions that historically limited field deployment. Early applications were primarily associated with moderate-temperature, moderate-salinity sandstone reservoirs with favorable injectivity and oil viscosities generally below approximately 150 mPa·s, consistent with the screening guidelines compiled by Taber et al. [69]. These criteria reflected concerns that increasing oil viscosity would create progressively unfavorable oil–water mobility ratios, requiring higher polymer viscosity, greater polymer concentrations, or additional pressure support to maintain displacement stability. However, Taber et al. [69] also emphasized that screening limits should be regarded as approximate guidelines rather than absolute technical boundaries. Accumulated field experience increasingly demonstrates that polymer flooding performance is governed by the integrated management of reservoir characteristics, injectivity, operational execution, and polymer qualification rather than strict adherence to a small number of screening thresholds [9,14,68]. The progressive expansion of polymer flooding from conventional sandstone applications toward offshore developments, heavy oil reservoirs, carbonate formations, high-temperature/high-salinity environments, and permeability-constrained reservoirs is summarized in Figure 4.

One long-standing assumption was that polymer flooding was primarily an onshore technology. This perception changed with the implementation of the Dalia and Camelia developments offshore Angola, which demonstrated successful polymer injection under large well spacing, subsea distribution systems, FPSO integration constraints, remote logistics, and injection water salinities of approximately 30,000 ppm TDS. These projects showed that offshore deployment challenges are principally related to polymer preparation, transport, viscosity preservation, logistics, and operational reliability rather than to any fundamental limitation of the polymer flooding process itself. Subsequent offshore developments and technology studies further expanded operating concepts for subsea and offshore chemical EOR applications [64,65].

Another long-standing assumption was that temperatures above approximately 80 °C, very-high-salinity brines, and carbonate reservoirs were generally unsuitable for polymer flooding. While these environments remain challenging, recent field programs have demonstrated injectivity qualification and polymer propagation under conditions previously considered beyond the practical operating envelope. Examples include carbonate injectivity pilots in Abu Dhabi operating at temperatures exceeding 120 °C and salinities above 260,000 ppm TDS, as well as multiple carbonate and sandstone projects in Kuwait utilizing ATBS-rich polymers under salinities exceeding 150,000 ppm TDS. Although many of these developments remain at the pilot or qualification stage, they demonstrate that polymer flooding can be technically evaluated and, in selected cases, successfully implemented far beyond the historical sandstone-dominated operating envelope [55,58,60].

Field experience has also challenged the assumption that polymer flooding requires low-salinity make-up water and very high reservoir permeability. Produced water polymer flooding projects in Kuwait demonstrated successful injection using produced waters with salinities approaching 166,000 ppm TDS, while pilot applications such as Palogrande-Cebu in Colombia demonstrated technically viable polymer propagation in permeability-constrained formations below 200 mD. These experiences highlight that water quality, permeability, and injectivity remain critical design variables but should not be interpreted as absolute exclusion criteria. Instead, project feasibility increasingly depends on integrated qualification of polymer chemistry, water treatment, molecular weight selection, reservoir heterogeneity, well architecture, and pressure management strategy (e.g., [57,82,83]).

Field deployment since the late 1990s has substantially exceeded the historical oil viscosity envelope. The field compilation of Delamaide [14] identifies nearly 20 polymer flood projects or pilots in heavy oil reservoirs, with numerous cases above 150 mPa·s and several involving oil viscosities from several hundred to several thousand mPa·s. Documented examples include Pelican Lake, Seal, Tambaredjo, East Bodo/Cosine, Patos Marinza, Cactus Lake, Nimr, Petrocedeño, and Milne Point. These applications are not limited to laboratory demonstrations; the reviewed cases include pilots, phased expansions, and large-scale developments. Pelican Lake is particularly important because it developed into the largest reported heavy oil polymer flood expansion, involving very viscous oil, horizontal wells, and injected polymer viscosity substantially below the oil viscosity. The collective evidence demonstrates that the approximate 150-mPa·s benchmark no longer represents a practical upper boundary for technical evaluation of polymer flooding (e.g., [4,14,67,70]).

Despite these successful heavy oil applications, oil viscosity remains a critical design consideration, and unfavorable mobility ratios cannot be disregarded. Rather, these applications show that endpoint mobility ratio alone may not adequately predict field performance. Several heavy oil projects achieved favorable production and water-cut responses while injecting polymer solutions with viscosities of only approximately 25–50 mPa·s, considerably below the oil viscosity. Proposed explanations include favorable shock front mobility, low endpoint water relative permeability, displacement through horizontal well patterns, viscous crossflow, and field-scale flow behavior not captured by simple one-dimensional displacement models. However, the relative contribution of these mechanisms remains unresolved, and strong performance should not be generalized across all heavy oil reservoirs. Technical and economic feasibility still depends on oil mobility at reservoir conditions, permeability, injectivity, allowable pressure gradient, well spacing, heterogeneity, polymer propagation, and project-specific economics. Consequently, high oil viscosity should be treated as an indicator of increasing design complexity rather than as an automatic exclusion criterion (e.g., [6,14,84]).

Collectively, these field experiences demonstrate that traditional screening criteria are most appropriately interpreted as indicators of technical complexity and project risk rather than immutable exclusion limits. However, not all extensions beyond traditional screening criteria have reached the same maturity. Heavy oil polymer flooding includes multiple pilots, expansions, and commercial-scale applications, whereas many high-temperature/high-salinity carbonate cases remain at the injectivity test or qualification stage. The evidence should therefore be interpreted according to project maturity and documentation quality. Across these environments, successful implementation depends on reservoir-specific mobility analysis, polymer qualification, injectivity management, well architecture, and operational discipline rather than polymer chemistry alone. Furthermore, confidence in reported incremental recovery increases greatly when production responses are supported by surveillance evidence such as tracer results, polymer breakthrough monitoring, pressure behavior, and history-matched reservoir studies (e.g., [9,14,68]).

## 5. Performance Across Global Projects

Interpretation of field performance should distinguish between pilot-scale results and mature commercial developments. Pilot projects are essential for evaluating injectivity, polymer propagation, and operational feasibility, but their recovery response may be constrained by limited injection duration, restricted areal extent, and interaction with surrounding waterflood operations. In contrast, mature full-field developments such as Daqing, Mangala, and Pelican Lake provide longer-term evidence of polymer flood performance under sustained reservoir-scale implementation. Accordingly, both project classes are valuable but provide different types of evidence and should not be interpreted equivalently when assessing ultimate recovery potential.

A synthesis of performance across the evaluated projects (dataset) is presented in Table 2 as a reservoir severity compilation. The table links temperature, salinity, polymer chemistry, and injectivity to reported field evidence, focusing on performance envelopes and implementation context. Polymer flooding performance is influenced by reservoir severity because increasing temperature and salinity impose additional constraints on polymer stability, viscosity retention, water quality requirements, and injectivity management. Reported field outcomes tend to become more variable under increasingly challenging reservoir conditions. However, differences in flood maturity, project scale, recovery definitions, and operational history limit direct comparison between cases. This observation is consistent with the literature indicating that success is governed by operability and robustness, rather than peak laboratory performance or polymer molecular weight alone (e.g., [2,7,10]).

Reported recovery performance varies across the severity classes summarized in Table 2; however, these trends should not be interpreted as a direct consequence of temperature or salinity alone. Recovery outcomes also depend on oil viscosity, mobility ratio, project maturity, injected polymer pore volume, reservoir architecture, surveillance quality, and project objectives. Consequently, Table 2 should be interpreted as a comparative synthesis of reported field evidence rather than a quantitative ranking of recovery potential. Importantly, the compiled data indicate that advances in polymer chemistry have expanded the applicability domain of polymer flooding but have not eliminated the fundamental physical limitations associated with polymer retention, degradation, and injectivity. These observations align with global field reviews suggesting that polymer flooding should be framed as a risk-managed mobility control process, rather than a viscosity-driven EOR technology (e.g., [7]).

Figure 5 provides a descriptive visualization of reservoir severity, showing how documented polymer flooding projects extend from mild and moderate conditions toward high-salinity and HSHT regimes as temperature and salinity increase. The figure illustrates the distribution of reported projects across the severity categories summarized in Table 2 and highlights the progressive expansion of polymer flooding into increasingly challenging environments. Recovery outcomes reflect project maturity, pilot design, injected pore volume, reservoir characteristics, mobility control, and development objectives in addition to polymer stability and injectivity considerations. Reported outcomes from higher-temperature and higher-salinity projects should therefore be interpreted in the context of project maturity, qualification objectives, and reporting basis. The distributions shown in Figure 5 should not be interpreted as normalized performance rankings because project maturity, recovery definitions, and injected polymer volumes varied substantially among cases. Instead, they illustrate the diversity of documented operating conditions represented within each reservoir severity category.

The severity classes proposed here are author-defined synthesis categories developed for comparative discussion of the reviewed dataset and should not be interpreted as industry standard screening criteria. Many carbonate HSHT projects included in the dataset were designed primarily as injectivity qualification tests rather than recovery pilots. Consequently, they cannot be interpreted as recovery performance benchmarks and should not be used to infer quantitative recovery limits associated with HSHT conditions (e.g., [55,60]).

### 5.1. Incremental Oil Recovery

Recovery performance comparisons should be interpreted with caution because the reviewed projects represent different stages of flood maturity, reporting practices, and performance metrics. The database includes observed and forecast recoveries, OOIP- and OIP-based recoveries, recovery factor (RF) improvements, displacement efficiency estimates, and ultimate recovery values. Consequently, reported recovery metrics are used in this review as indicators of documented field performance rather than as directly comparable measures of technology effectiveness across all projects. The database itself contains a specific comparability warning because recovery entries combine different recovery definitions and evidence types.

Reported recovery metrics must therefore be interpreted in relation to oil viscosity, initial mobility conditions, injection timing, project maturity, and the selected recovery baseline. In conventional light- to medium-oil reservoirs, including Daqing and Mangala, polymer flooding is primarily applied to improve an adverse or moderately unfavorable water–oil mobility ratio and enhance volumetric sweep following waterflooding. In the reviewed database, Daqing reports approximately 12–25% recovery improvement, whereas Mangala reports an approximately 7% increase in recovery factor relative to waterflood performance. Although these values are often cited as benchmarks, they are not directly comparable because they reflect different recovery definitions, operating conditions, and project histories (e.g., [5,71]).

Heavy oil reservoirs represent a distinct mobility context. At Pelican Lake, polymer flooding remains effective despite strongly unfavorable oil–water mobility conditions, with reported mobility ratios exceeding 10. Rather than creating a stable displacement front, polymer injection primarily reduces aqueous mobility and suppresses channelized water flow, thereby improving sweep efficiency [4,14]. Reported performance varies according to project scale and reporting basis. The database records approximately 7–10% OOIP incremental recovery for field applications, approximately 26% OOIP for pilot areas, and estimated ultimate recoveries ranging from 20 to 38% OOIP in polymer-treated developments. These values should not be aggregated because they represent different stages of implementation and different performance metrics (e.g., [4,14,67,84,85,86]).

These heavy oil experiences demonstrate that achieving a mobility ratio of unity is not necessarily required for favorable field performance. Partial reduction in a strongly unfavorable mobility condition may substantially improve sweep efficiency and oil recovery under suitable reservoir and operational conditions. However, these outcomes should not be generalized across all heavy oil reservoirs because performance remains influenced by injectivity, reservoir architecture, heterogeneity, well configuration, pressure constraints, polymer propagation, and operational execution [4,14]. For comparative interpretation, the reviewed projects can be grouped into three broad mobility contexts. First, heavy oil reservoirs such as Pelican Lake, Milne Point, East Bodo, Tambaredjo, Nimr, and Patos-Marinza typically begin with low recovery under primary production or conventional waterflooding because of severe mobility contrast. In these cases, polymer flooding seeks to control water mobility and improve displacement efficiency, even when favorable endpoint mobility ratios are not achieved [6,14]. Second, light- to medium-oil developments such as Daqing, Mangala, Kalamkas, and Diadema primarily use polymer flooding to improve sweep efficiency within mature waterfloods, with performance strongly influenced by heterogeneity, polymer bank size, injection strategy, and surveillance-driven reservoir management [5,14]. Third, where pre-polymer mobility conditions are already relatively favorable, the incremental benefit of mobility control may be smaller, and performance depends more strongly on remaining sweep inefficiencies and reservoir heterogeneity. These categories remain interpretive because viscosity alone does not determine polymer flood success.

#### Design Rationale Behind Polymer Flooding Strategies

Field applications demonstrate that polymer injection strategies are selected according to reservoir conditions, operational constraints, and project objectives rather than following a universal design. Higher polymer concentrations are generally adopted when stronger mobility control is required, particularly in reservoirs with unfavorable mobility ratios, higher oil viscosities, or pronounced heterogeneity. Lower concentrations may be selected when injectivity constraints or economic considerations dominate. Tapered concentration schemes are frequently implemented to achieve early mobility control while reducing polymer consumption during later stages of the flood. Similarly, larger polymer slugs are designed to maintain sweep efficiency improvements over a greater reservoir volume, whereas smaller slugs are often used in pilot projects to reduce investment and evaluate field response.

The timing of polymer deployment also influences strategy selection. Secondary polymer floods seek to establish favorable mobility conditions before significant channel development occurs, thereby preserving sweep efficiency from the outset. In contrast, tertiary polymer floods are designed to improve sweep in reservoirs already affected by water channeling and bypassed oil accumulation. In some cases, cyclic injection schemes are applied to address injectivity limitations, improve operational flexibility, or evaluate reservoir response before continuous polymer injection. These field experiences demonstrate that injection concentration, slug size, injection schedule, and deployment timing should be tailored to reservoir architecture, fluid properties, and project objectives rather than applied uniformly across different assets.

The relationship between reported recovery metrics and reservoir operating conditions is illustrated in Figure 6. The compiled dataset shows substantial variability across the full range of reported temperatures and salinities, without indicating a simple monotonic relationship between the reported recovery metric and either variable alone. This observation is consistent with field evidence suggesting that polymer flood performance reflects the combined influence of reservoir quality, oil viscosity, heterogeneity, mobility ratio, project maturity, injectivity management, surveillance quality, injected polymer pore volume, and operational execution rather than temperature or salinity alone (e.g., [56,73,74]). Likewise, the dispersion observed among projects operating under similar temperature and salinity conditions supports previous findings that sustained polymer propagation and effective mobility control are generally more important to field performance than peak injected viscosity alone [7,87].

Only projects reporting numerical recovery, temperature, and salinity values were included in Figure 6. Where numerical ranges were reported, midpoint values were used solely for graphical positioning and did not replace the original values retained in the database. Because recovery values retain their original reporting basis, the figure should be interpreted as a descriptive comparison of reported field evidence rather than a quantitative ranking of polymer flood effectiveness.

### 5.2. Polymer Retention and Adsorption

Polymer retention results from a combination of adsorption onto rock surfaces and mechanical entrapment within porous media. Its magnitude is governed by interacting factors, including lithology, mineralogy, permeability, polymer chemistry, salinity, hardness, oil saturation, and wettability. Adsorption is generally promoted by electrostatic interactions, hydrogen bonding, and van der Waals forces, whereas mechanical retention becomes increasingly important in low-permeability formations and pore systems with significant geometric restrictions [9,88,89]. However, the relative contributions of adsorption, mechanical entrapment, hydrodynamic retention, inaccessible flow pathways, and rock heterogeneity remain incompletely resolved. Consequently, reported retention values should be interpreted as outcomes measured under specific experimental or field conditions rather than as universally transferable reservoir properties.

Mineralogy can exert an important influence on retention behavior. Clay-rich rocks may exhibit higher retention because of their greater specific surface area and stronger polymer–surface interactions. Seright and Wang [9] (2023) reported retention values exceeding 200 µg/g-rock in some clay-rich systems, whereas lower values have been documented under other mineralogical and brine conditions. In carbonate reservoirs, retention is additionally affected by surface charge, which depends on brine salinity, hardness, and pH. High-salinity and high-hardness brines may strengthen interactions between anionic polymers and carbonate surfaces, although contrasting trends have been reported depending on polymer chemistry, rock mineralogy, and water composition [9,90,91].

Retention reporting remains incomplete across the field project database. Of the 54 reviewed entries, 23 contain some form of retention information (approximately 43% of the database), whereas 31 do not report a retention value. Moreover, several of the reported entries are qualitative, inferred, or expressed on a basis that does not permit direct comparison. Therefore, only projects providing explicit numerical retention values in µg/g-rock were included in Figure 7. Within this numerical subset, reported retention values or ranges extend from approximately 10 to 65 µg/g-rock. The sandstone entries include both HPAM- and ATBS-modified systems, with project-level ranges commonly beginning between approximately 10 and 20 µg/g-rock and extending to upper reported values between approximately 30 and 65 µg/g-rock. The two explicitly classified carbonate entries included in the numerical subset, Mauddud DRCC and Minagish Oolite, report ranges of approximately 10–30 and 10–40 µg/g-rock, respectively. The database therefore does not support a general conclusion that carbonate projects consistently exhibit higher retention than sandstone projects. The available carbonate subset is also too limited to establish a statistically defensible lithology-dependent distribution.

The numerical data likewise do not establish that ATBS-containing polymers consistently reduce retention relative to conventional HPAM. Most projects reporting explicit numerical retention are recent ATBS-HPAM pilots in Kuwait, while comparatively few conventional HPAM projects provide directly comparable values. The observed distribution consequently reflects differences in project coverage, reservoir setting, polymer formulation, and reporting practice as well as potential polymer–rock interactions. Comparisons among polymer families should therefore be made only where measurements were obtained under sufficiently similar mineralogical, brine, saturation, and experimental conditions.

The retention values summarized in Figure 7 should also be interpreted in relation to measurement methodology. Sebastian et al. [39] demonstrated that dynamic retention calculated for the same ATBS-based polymer–limestone system varied with the analytical method used to quantify effluent polymer concentration. Measurements based on UV spectroscopy, viscosity correlations, and TOC-TN analysis produced different retention estimates under comparable coreflood conditions, and uncertainty in polymer concentration measurements propagated directly into the calculated retention values. Differences in analytical methodology, experimental design, oil saturation, sampling procedure, and uncertainty treatment must therefore be considered when comparing reported results. Retention ranges are most appropriately treated as project- and method-specific observations rather than universal adsorption constants.

Despite these uncertainties, polymer retention remains an important design parameter because excessive retention can delay polymer propagation, increase polymer requirements, alter the effective polymer bank size, and adversely affect project economics. The field database also identifies retention as a major reporting and comparability gap rather than demonstrating a universal relationship between retention and project outcome. Retention should therefore be evaluated together with injectivity, polymer stability, propagation behavior, water quality, reservoir mineralogy, and project-specific operating conditions rather than as a stand-alone screening criterion [9].

### 5.3. Viscosity Stability and Degradation

Polymer flooding requires the selected polymer to maintain sufficient viscosity and chemical integrity throughout the anticipated reservoir residence time under project-specific brine composition, temperature, oxygen exposure, and mechanical shear conditions. Accordingly, temperature, salinity, and hardness should not be interpreted as universal screening limits but rather as qualification conditions against which candidate polymers must be evaluated. Viscosity stability is polymer-specific and depends on the interaction of molecular architecture, brine chemistry, divalent ion concentration, temperature, oxygen control, mechanical exposure, and residence time rather than on a single salinity-temperature threshold [21,22].

Conventional HPAM may become unsuitable in some high-temperature or hard-brine environments because progressive hydrolysis can increase sensitivity to divalent ions and promote viscosity loss or precipitation. In contrast, ATBS-rich, sulfonated, and other thermally stable polymers can extend the applicable operating envelope when their composition is matched to reservoir brine chemistry and long-term exposure conditions [21,22]. Field observations from the Mangala polymer flood illustrate this polymer-specific qualification challenge. Extended reservoir exposure was associated with increased hydrolysis, viscosity reduction, cloud point depression, and precipitation tendency, motivating the evaluation of alternative ATBS-containing formulations rather than establishing a universal temperature limit for polymer flooding [61,92,93]. Similar field observations reported in Aishwariya, Matzen, Palogrande-Cebu, Kalamkas, and Sirikit demonstrate that viscosity losses may arise from hydrolysis, oxygen exposure, iron contamination, microbial activity, or unsuitable handling conditions rather than temperature alone.

Significant viscosity degradation should not be regarded as an expected outcome of properly designed polymer flood facilities. Several projects in the reviewed database, including Daqing, Milne Point, Marmul, Minagish Oolite, Uzen, and multiple Kuwait pilots, reported stable or acceptable viscosity performance under field conditions when polymer selection, water treatment, oxygen control, and handling procedures were appropriately managed. Conversely, documented degradation events were generally linked to project-specific operational issues or polymer–reservoir incompatibilities rather than unavoidable surface-handling effects [70,72,94].

#### Long-Term Polymer Stability During Reservoir Residence

Field performance is influenced not only by initial polymer viscosity but also by the evolution of polymer properties during prolonged reservoir residence. Progressive hydrolysis can alter polymer charge density and thereby influence viscosity stability, adsorption behavior, injectivity, flow resistance, and long-term mobility control [21,22]. Conventional HPAM systems are generally more susceptible to hydrolysis at elevated temperatures, whereas ATBS-containing and sulfonated polymers are designed to provide improved thermal and chemical stability.

Field observations from Mangala indicate that extended reservoir exposure may be associated with viscosity reduction, cloud point depression, changes in rheological behavior, and increased precipitation tendency for certain polymer formulations [61,85,92,93]. Similar observations from other projects indicate that polymer properties may evolve over the operational life of a flood and that short-duration laboratory testing may not always capture long-term reservoir behavior. These experiences emphasize the importance of reservoir-specific qualification, long-duration stability testing, and periodic evaluation of polymer performance under representative operating conditions.

Because polymer properties may change throughout the life of a flood, long-term project management benefits from monitoring water quality, produced polymer concentration, pressure behavior, and viscosity retention. Such information can help distinguish reservoir effects from chemical aging, hydrolysis, operational degradation, or compatibility issues while improving confidence in long-term mobility control performance [21,22].

### 5.4. Injectivity Physics and Mechanisms

Injectivity remains a principal technical challenge in polymer flooding, particularly in low-permeability and heterogeneous reservoirs. Recent carbonate field evidence indicates that injectivity behavior is controlled by the combined effects of polymer bank propagation, resistance factor development, reservoir heterogeneity, inaccessible pore volume, relative permeability, and operational pressure management rather than by a single plugging mechanism. Carbonate reservoirs commonly exhibit complex pore structures and large permeability contrasts that can cause substantial flow redistribution during polymer injection. Middle Eastern carbonate injectivity tests documented progressive declines in injectivity index during polymer bank establishment, but these responses were interpreted in relation to mobility control development, water quality, and reservoir heterogeneity rather than solely as irreversible polymer plugging [55,60]. Retention and permeability reduction measurements should similarly be interpreted within their reservoir and measurement context because observed injectivity combines polymer transport, adsorption, inaccessible pore volume, relative permeability, and operating constraints [39].

Field injectivity is not controlled solely by polymer viscosity or near-wellbore retention. It also depends on reservoir flow capacity, oil–water relative permeability, fracture behavior, and the overall displacement mobility. In reservoirs containing viscous oil, the oil phase can remain a major contributor to flow resistance, causing injection pressure to depend on the polymer–oil mobility condition until substantial water or polymer breakthrough occurs [9,70]. Consequently, injectivity cannot be predicted reliably from bulk polymer rheology alone and should be evaluated using representative relative permeability, pressure gradient, and polymer propagation data.

Excessive injection pressure may initiate or extend fractures, particularly in lower-permeability formations or when higher-viscosity polymers are injected at aggressive rates. Controlled fracture-assisted injection has supported polymer throughput in some projects, including Tambaredjo, whereas uncontrolled fracture growth can alter flow distribution and increase channeling risk [84]. Fracture development should therefore be considered a project-specific design variable with both potential benefits and risks rather than solely as an operational failure mode.

Laboratory studies also indicate that injectivity may be influenced more strongly by high-molecular-weight polymer fractions, microgels, and other solution quality limitations than by rheometer viscosity alone. Polymers with broader molecular weight distributions can exhibit greater resistance factors and slower propagation despite similar bulk viscosities, emphasizing the importance of polymer quality, filtration, and molecular weight selection [90,95]. The principal injectivity observations represented in the reviewed database are summarized in Table 3 and Figure 8.

### 5.5. Operational Management and Surveillance

Pressure increases during polymer injection should not be interpreted automatically as evidence of irreversible injectivity impairment. As polymer banks propagate and mobility control develops, injection pressure commonly increases relative to waterflooding, while injectivity responses range from maintained to operationally challenging depending on reservoir conditions and project execution. Field experience shows that injectivity performance is influenced by factors such as polymer molecular weight selection, water quality, near-wellbore condition, fracture management, and facilities performance rather than salinity or temperature alone (e.g., [7,56,71,86]). The reviewed database identifies injectivity and pressure management as the most frequently reported operational issue, followed by water channeling, polymer stability, water quality, and facilities-related concerns. Therefore, injectivity outcomes should be interpreted within the broader operational context of polymer propagation, reservoir heterogeneity, and mobility control development rather than as stand-alone indicators of project success or failure [7,96]. The distribution of reported injectivity outcomes is presented in Figure 8, while Figure 9 summarizes the recurring operational issues identified across the reviewed database.

### 5.6. Water Quality Requirements

Water quality is a critical enabler of polymer flood performance because it affects polymer stability, injectivity, degradation behavior, filtration, facility operability, and long-term reliability. Conventional polymer flooding primarily focuses on managing oxygen, iron, suspended solids, oil contamination, polymer solution quality, and polymer–brine compatibility [94,96]. In addition to chemical contaminants, microbial activity can contribute to polymer degradation, biofilm formation, filtration problems, and injectivity decline if not adequately controlled through water treatment and biocide programs. The database records water quality or chemistry issues in 14 projects, confirming that these concerns are important but are not reported uniformly across the project population.

The reviewed projects demonstrate that conventional polymer flooding has been evaluated across a wide salinity range. Reported cases extend from low-salinity source waters to brines exceeding 250,000 ppm TDS. For example, UNLF used produced water at approximately 166,000 ppm TDS, while the Abu Dhabi carbonate injectivity tests reported approximately 260,000–264,000 ppm TDS. Several Kuwait pilots also report salinity ranges exceeding 150,000 ppm TDS. These observations demonstrate field qualification under high-salinity conditions but do not define universal water quality limits [56,60].

Conventional polymer and alkali–polymer flooding differ in the water quality issues governing their design. AP flooding introduces additional sensitivity to divalent ions because alkali can promote precipitation, scaling, and process instability. AP-specific hardness and treatment targets should therefore not be applied as generic requirements for conventional polymer flooding [97,98]. Table 4 accordingly separates database-supported conventional polymer observations from project-specific AP treatment examples.

### 5.7. Economic Performance

The economic performance of polymer flooding (Table 5) is governed by the combined effects of incremental oil recovery, production acceleration, polymer utilization efficiency, injectivity sustainability, facility requirements, and reductions in water-handling costs rather than ultimate recovery alone. The reviewed field applications report a wide range of recovery outcomes, including approximately 4% incremental OOIP at Baytex, approximately 7.5% field-wide incremental recovery at Kalamkas, 12–25% at Daqing, and 7–10% field recovery with ultimate estimates of 20–38% OOIP at Pelican Lake. However, these values are not directly comparable because the published data combine observed and forecast results, different OOIP, OIP, recovery factor, and displacement efficiency bases, absolute production volumes, and project-specific reporting periods. Therefore, the values summarized in Table 5 are presented as individual field examples rather than as a statistically representative industry range [7,84,87].

Economic outcomes are highly sensitive to polymer utilization efficiency because polymer procurement and preparation can represent a major variable operating cost component. At Baytex, polymer flooding OPEX was reported to be approximately twice that of waterflooding, with polymer accounting for approximately 70% of the incremental cost. However, project profitability is also strongly influenced by injectivity, well costs, facility investment, produced water treatment, project scale, oil price, and the timing of incremental oil response. Published field evaluations illustrate the diversity of reported economic indicators. Kalamkas reported an estimated NPV of approximately USD 695 million, a profitability index of approximately 1.6, approximately USD 34.7 million in OPEX savings, and around 60 MMbbl of water savings. Uzen reported a modeled profitability index of 1.93, payback in Year 3, and a polymer utility factor of 50.6 t oil/t polymer, whereas East-Messoyakhskoye reported an NPV of approximately RUB 125.4 million, a profitability index of 1.40, and approximately 43 t oil/t polymer. These values demonstrate potential commercial viability but should not be compared directly because they reflect different currencies, economic assumptions, cost boundaries, project maturities, and reporting dates [7,84,98].

Other field cases provide complementary unit cost and optimization indicators. Courtenay/Châteaurenard reported approximately 265,000 bbl of additional oil at a total cost of about USD 12/bbl, while Minagish Oolite reported unit technical costs of approximately USD 7–23/bbl and Medicine Hat reported a finding and development cost of approximately USD 20/BOE. At Marmul, optimization of the polymer slug to approximately 0.5–0.7 PV was estimated to reduce polymer consumption by 26–36%. These examples indicate that economic performance depends not only on incremental recovery but also on the efficiency with which polymer, wells, facilities, and injection capacity are converted into additional production [1,7,86].

Water-handling reduction can provide additional value by lowering lifting, treatment, disposal, and energy requirements. Kalamkas reported approximately USD 34.7 million in OPEX savings associated with around 60 MMbbl of water savings, while the Captain development linked polymer injection to improved oil production, reduced water handling, and the economic viability of an otherwise negative-NPV infill opportunity [90]. The Yariguí-Cantagallo thermally active polymer campaigns similarly reported lower water-handling requirements, a 6% improvement in energy intensity, and a reduction in carbon intensity from 35.0 to 33.5 kg CO_2_/BO [91,92,93,94,95,96,97,98,99,100]. Nevertheless, the monetary value of these benefits remains project-specific because it depends on water treatment capacity, disposal costs, energy demand, facility ownership, and baseline operating conditions [7,87].

Field experience indicates that economic underperformance is more frequently associated with injectivity deterioration, excessive polymer consumption, delayed or spatially uneven oil response, water quality limitations, polymer degradation, conformance problems, or facility constraints than with an absence of remaining oil. In the updated database, injectivity or pressure-related issues were identified in 23 of the 54 project records, water or channeling issues in 21, polymer degradation or stability issues in 15, and facility or logistics constraints in 10. These counts represent reported operational themes rather than failure rates because the completeness and level of detail vary among publications. Consequently, successful polymer developments are typically implemented through staged appraisal, injectivity testing, pilot surveillance, polymer quality control, and progressive scale-up to reduce both technical and economic uncertainty [7,88,101].

As shown in Figure 10, polymer selection generally becomes more specialized with increasing reservoir severity. Conventional HPAM or generically reported polymer systems dominate the mild category, whereas all nine projects classified as high salinity use ATBS-containing or other specialty polymers. The four HSHT cases comprise three ATBS-containing or specialty polymer applications and one particle, microgel, or hybrid system. However, 21 of the 54 project records lack sufficient numerical salinity or temperature information for consistent classification and are therefore presented separately as “Insufficient data.” The resulting distribution reflects the industry’s adaptation of polymer chemistry to reservoir conditions, but it should not be interpreted as a universal technology hierarchy or as evidence of comparative economic performance [1,7,84].

Detailed economic disclosures remain relatively uncommon in the public polymer flooding literature. Information on facility CAPEX, polymer cost assumptions, polymer consumption per incremental barrel, operating expenditures, oil price assumptions, and project cash flows is often proprietary or reported inconsistently. Differences in accounting practices, facility ownership, project maturity, economic dates, and reporting scope further complicate direct comparisons of project profitability. Accordingly, Table 5 summarizes reported economic indicators, project-specific outcomes, and operational lessons without attempting to calculate average costs or rank projects economically. The available evidence nevertheless consistently identifies polymer utilization efficiency, injectivity, facility investment, well costs, water handling, project scale, and the timing of incremental oil response as principal economic drivers [7,84,98].

## 6. Success Factors, Failure Modes, and Applicability Limits

Polymer flooding outcomes vary widely even among projects using similar polymer families. The reviewed cases indicate that performance depends on the combined effects of reservoir architecture, mobility conditions, injectivity, water and solution quality, facility reliability, surveillance, and operational response. Polymer chemistry defines the conditions under which viscosity and compatibility can be maintained, but it cannot compensate for inadequate flow capacity, severe channeling, insufficient pressure margin, or inconsistent field execution [3,7,9].

The operational issue coding of the 54-project database identified injectivity or pressure concerns in 23 records, water or channeling issues in 21, polymer degradation or stability issues in 15, water quality or chemistry issues in 14, and facility or logistics constraints in 10. These themes overlap within individual projects and represent the frequency with which issues were documented, not mutually exclusive failure causes or industry-wide failure rates. Figure 11 therefore shows that injectivity management and sweep control are the most frequently reported implementation concerns, while polymer stability, water quality, and surface system reliability remain material but less uniformly documented constraints.

Figure 11 should not be interpreted to imply that all pressure increases represent operational problems. In polymer flooding, injection pressure commonly rises relative to waterflooding as polymer banks propagate, mobility control develops, and resistance factors increase within the reservoir. A pressure increase may therefore be a normal consequence of establishing mobility control rather than evidence of injectivity impairment. Conversely, persistent pressure escalation accompanied by declining injectivity, reduced injection rates, poor polymer propagation, abnormal pressure-transient behavior, or increasing stimulation frequency may indicate operational concerns requiring investigation. Because reservoir permeability, completion design, fracture behavior, polymer properties, water quality, and operating constraints vary considerably among projects, the reviewed database does not support universal pressure thresholds distinguishing normal and abnormal responses. Instead, pressure behavior should be evaluated together with injectivity index, polymer concentration, water quality, production response, tracer results, and surveillance data. Common mitigation measures reported in the literature include adjustment of polymer molecular weight or concentration, injection rate optimization, water quality improvement, stimulation, conformance treatments, fracture management strategies, and surveillance-guided pattern reallocation.

The database also highlights major evidence limitations. Temperature is reported for 46 of 54 entries, salinity for 44, recovery-related outcomes for 44, oil viscosity for 37, and polymer retention for only 23. Recovery information combines observed and forecast outcomes, different recovery bases, absolute production volumes, and qualitative statements. Consequently, the reported frequencies and project outcomes should be interpreted as benchmarking evidence rather than statistically representative failure probabilities or directly comparable performance rankings.

Water quality and chemistry requirements should likewise be treated as project-specific operating envelopes rather than universal specifications. Required control of dissolved oxygen, iron, suspended solids, oil contamination, bacteria, polymer impurities, and brine compatibility depends on polymer chemistry, source water composition, reservoir temperature, facility configuration, and the selected produced water strategy. Alkaline polymer water treatment criteria should not be transferred directly to conventional polymer flooding because alkali introduces additional hardness, precipitation, scaling, and process stability constraints [94,96,97].

### 6.1. Reservoir and Operating Envelopes

Field experience demonstrates that polymer flood feasibility is governed by coupled reservoir and operating conditions rather than isolated screening thresholds. Permeability, pore throat architecture, oil mobility, heterogeneity, well configuration, polymer molecular weight and concentration, injection rate, relative permeability, and pressure relative to fracture initiation jointly determine injectivity and propagation [9,68,72].

The updated database includes maintained, manageable, and challenging injectivity responses across overlapping permeability and salinity ranges. Polymer propagation has been demonstrated below 200 mD in selected projects, while injectivity problems have also occurred in reservoirs with substantially higher permeability. Permeability and salinity should therefore be used as qualification variables rather than deterministic acceptance criteria. Project-specific testing should establish whether the required polymer concentration and injection rate can be sustained within the available pressure window and with an acceptable flow distribution.

Heterogeneity and fracture behavior require equally careful interpretation. Controlled fracture-assisted injection has maintained polymer throughput in projects such as Tambaredjo, whereas poorly constrained fracture growth or high-permeability pathways can promote channeling and early breakthrough. A pressure increase during polymer injection does not by itself demonstrate formation damage because pressure may rise as the polymer bank propagates and resistance develops. Conversely, apparently high injectivity may reflect preferential fracture or thief zone flow rather than effective matrix contact [7,73].

Reservoir risks vary considerably among reservoir types and should be evaluated during project qualification. In sandstone reservoirs, the principal risks generally relate to injectivity limitations, polymer degradation, channeling, excessive retention, water quality issues, and operational execution. Carbonate reservoirs introduce additional uncertainty because of greater heterogeneity, complex pore systems, permeability contrasts, fracture-controlled flow, and less extensive commercial field experience. Several carbonate projects reviewed in this study successfully demonstrated polymer injectivity and propagation under challenging conditions; however, injectivity decline, uncertain sweep development, fracture-dominated flow paths, and limited full-field recovery validation remain important technical risks. Consequently, carbonate polymer flood projects typically require more extensive injectivity qualification, surveillance, and uncertainty management than conventional sandstone applications.

Reservoir readiness is therefore better assessed through an integrated operating envelope that combines injectivity, fracture pressure margin, reservoir connectivity, mobility conditions, polymer propagation, and surveillance-supported flow distribution. Published injection rate or slug-size ranges may provide useful project analogs, but they should not be applied as universal success thresholds.

### 6.2. Polymer Formulation and Chemistry Constraints

Polymer chemistry governs viscosity generation, thermal and chemical stability, retention, and compatibility with the selected water. Conventional HPAM remains technically and economically attractive under qualified conditions, while ATBS-containing and other sulfonated polymers extend stability into harder, more saline, or higher-temperature environments [21,22]. Nevertheless, the database shows that successful injection under harsh conditions remains more widely demonstrated than full-field commercial recovery. Chemistry extends the qualification envelope but does not remove constraints imposed by permeability, pressure capacity, heterogeneity, facilities, or economics.

Qualification should eliminate formulations with inadequate dissolution, unstable viscosity, poor filtration behavior, or unacceptable injectivity before extended testing. However, slow dissolution, fisheyes, microgels, incomplete hydration, and external contamination should be distinguished from intrinsic molecular weight-distribution effects because many solution quality problems can be mitigated through preparation, hydration, filtration, and quality control measures [68].

Polymer molecular weight distribution provides additional information beyond nominal molecular weight and bulk viscosity. Laboratory core-flood, Field-Flow Fractionation, and selected produced polymer studies indicate that substantial high-molecular-weight tails or oversized fractions can contribute disproportionately to filtration, mechanical entrapment, retention, delayed propagation, and near-wellbore flow resistance, particularly in permeability-constrained media [90,95]. These observations are relevant to polymer selection but do not establish a universal field ranking of narrow and broad molecular weight distributions.

Accordingly, molecular weight distribution should complement, rather than replace, solution quality testing, rheology, filtration assessment, injectivity testing, and representative core flooding. Advanced analytical methods such as field flow fractionation are useful diagnostic tools when molecular-size effects must be resolved, but they are not required for every field-screening program. The practical interpretation of the available evidence is summarized in Table 6.

To better understand Table 6, the observations summarized are derived primarily from laboratory core-flood experiments, FFF measurements, and selected field observations. The reported trends are illustrative rather than universal because injectivity and propagation also depend on permeability, pore throat architecture, reservoir heterogeneity, polymer chemistry and concentration, water and solution quality, injection rate, operating pressure, completion design, and fracture behavior. Operational problems caused by fisheyes, microgels, incompletely hydrated particles, or external contamination should not be attributed automatically to intrinsic MWD effects.

With regard to residual resistance factor (RRF) interpretation, RRF is an experiment-specific indicator of post-polymer flow alteration rather than a directly transferable measure of formation damage or field-scale injectivity impairment. Reported RRF values depend on core properties, polymer history, injection rate, pressure stabilization criteria, post-flush duration, and calculation methodology. The measured response may include contributions from adsorption, mechanical retention, inaccessible flow pathways, and non-Darcy effects associated with retained polymer structures [9,102].

### 6.3. Operational Execution and Surveillance

The documented cases associate sustained performance with reliable water treatment, consistent polymer preparation, controlled mechanical exposure, injectivity management, and surveillance-supported adjustment. These elements should be treated as an integrated operating system because a deviation in one area can affect polymer viscosity, injection pressure, reservoir distribution, produced water handling, and recovery response [7,98,102].

Surface facilities must consistently deliver the specified polymer concentration and solution quality. Mixing energy, hydration and maturation time, filtration performance, metering, storage, transport, and choke or pump configuration should therefore be monitored against field-qualified limits. Both powder and liquid polymer products can be technically viable, but the selected form must be evaluated against active-polymer content, preparation quality, injectivity, facility footprint, logistics, and life cycle cost rather than assumed to be intrinsically superior [33,34,36].

Surveillance should integrate injection pressure and rate, injectivity index, polymer concentration and viscosity, produced polymer response, water cut, oil rate, voidage replacement, tracer evidence where available, and facility performance. Individual indicators are not diagnostic by themselves. For example, increasing pressure may reflect polymer bank establishment or injectivity deterioration, while early polymer production may indicate fractures, thief zones, or stronger-than-expected connectivity. Integrated interpretation is required before changing polymer concentration, injection rate, well allocation, or conformance strategy [9,73,102].

### 6.4. Dominant Failure Modes

The database does not support the statement that most polymer flood failures originate from a single mechanism. Instead, it identifies several recurring and interacting failure modes:

- Injectivity and pressure limitations: inadequate pressure margin, unsuitable molecular weight or concentration selection, near-wellbore skin, solution quality problems, and changing fracture behavior can restrict polymer delivery.
- Channeling and poor conformance: fractures, thief zones, strong permeability contrasts, or aquifer communication can accelerate breakthrough and reduce contacted reservoir volume.
- Polymer degradation or instability: oxygen, iron, mechanical shear, microbial activity, thermal exposure, and incompatible brines can reduce effective viscosity or cause precipitation.
- Water quality, microbial activity, and solution quality deficiencies: suspended solids, oil contamination, dissolved oxygen, bacterial contamination, biofilm development, fisheyes, microgels, and incomplete hydration can increase filtration requirements, promote polymer degradation, contribute to injectivity impairment, and reduce effective polymer performance. Several field applications reported the need for biocide treatment and microbial control as part of maintaining long-term injectivity and viscosity stability [94,102].
- Facility and logistics constraints: preparation system downtime, inadequate maturation, equipment failures, storage and transport restrictions, and produced polymer handling can interrupt or degrade injection.

These mechanisms frequently coexist; for example, water quality deterioration may first appear as a decline in injectivity. Excessive viscosity or molecular weight may increase pressure and promote fracture extension rather than simple plugging. Failure diagnosis should therefore separate the observed symptom from the underlying reservoir, polymer, facility, or operating cause. The updated evidence also does not justify universal statements that successful floods require more than 0.5 PV of polymer, that slugs below 20–30% PV fail, or that failure probability increases sharply after channel establishment. Polymer volume, concentration, tapering, injection rate, and economic duration must be optimized for each reservoir. Published experience supports the need for a sufficiently sustained polymer bank, but no single pore volume threshold can be applied across all project types [6,52].

### 6.5. Frontier and Hybrid Applications

Frontier applications include offshore, high-salinity, high-temperature, carbonate, low-permeability, and operationally constrained reservoirs. The database demonstrates that polymer injection can be technically qualified under conditions extending to approximately 124 °C and 270,000 ppm TDS. However, many of the harshest cases remain injectivity tests, pilots, or technology qualification programs rather than mature commercial recovery projects. Technical injectivity should therefore not be interpreted as evidence of full-field recovery or economic scalability.

Offshore deployment imposes additional constraints related to footprint, polymer storage and transport, preparation capacity, mechanical degradation, subsea distribution, produced water handling, and system reliability [64,65,66]. Encapsulated, delayed viscosity, associative, particle-based, and crosslinked systems may help address specific injectivity or conformance limitations, but their value should be assessed against simpler qualified polymers and the full facility and operating requirements [63,68].

Future applicability should therefore be judged through staged evidence: representative laboratory qualification, field injectivity and propagation, surveillance-supported pilot response, reliable facilities, and project-specific economics. Hybrid or advanced chemistries are best treated as targeted risk-mitigation options rather than universal extensions of polymer flooding.

## 7. Integrated Field Lessons Learned (2005–2025)

More than two decades of pilots and commercial deployments have generated a substantial body of operational evidence across reservoir types, geographic regions, and development settings. The reviewed database contains 54 documented project records, including 39 pilot or trial projects and 11 full-field or commercial implementations. Comparison of these case histories reveals recurring operational themes that distinguish more successful deployments from projects that underperformed or encountered scaling challenges. The following synthesis focuses on field-observed practices that consistently supported performance, recurring causes of underperformance, and operational indicators that warrant early intervention.

### 7.1. What Consistently Worked

#### 7.1.1. Reservoir Selection, Deployment Strategy, and Project Qualification

- Several successful projects reported polymer deployment before substantial reservoir depletion [3,4,102,103]. This suggests that earlier implementation may preserve sweep efficiency, delay channel development, and improve polymer utilization efficiency. However, the reviewed database also contains successful tertiary polymer floods, indicating that implementation timing should be optimized according to remaining recovery potential, waterflood maturity, and reservoir-specific operating conditions rather than a universal water-cut or depletion threshold.
- Secondary polymer floods preserve reservoir connectivity and may reduce polymer consumption compared with later deployment strategies [3].
- Successful developments commonly progressed through laboratory qualification, injectivity testing, monitored pilot operation, and progressive field expansion [68,98]. This approach allowed polymer selection, injection strategy, facility design, and surveillance programs to be adjusted before larger capital commitments were made. Although not a mandatory development pathway, staged qualification was a recurring characteristic of many mature projects.
- Disciplined laboratory workflows accelerated deployment by eliminating unstable polymers and unsuitable formulations early in the evaluation process [70]. Reservoir-specific qualification of polymer chemistry, molecular weight, salinity tolerance, water composition, oxygen sensitivity, and long-term stability generally proved more important than reliance on generic screening criteria. Successful applications were reported across a wide range of reservoir conditions when injectivity, stability, and water compatibility were qualified together [55,56,82].

#### 7.1.2. Injectivity and Mobility Control Management

- Projects prioritizing injectivity over maximum viscosity achieved larger contacted reservoir volumes [68]. This strategy frequently improved both technical and economic outcomes by maintaining sustainable injection rates within available pressure limits.
- Polymer molecular weight matched to reservoir permeability helped preserve injectivity and reduce near-wellbore impairment [72]. Similarly, projects employing adequately sized polymer banks generally reported more favorable recovery outcomes than projects using undersized polymer slugs. Field experience indicates that polymer bank size, concentration, and injection duration must be sufficient to establish and maintain mobility control and sweep improvement. However, the present database does not support a universal pore-volume threshold applicable to all reservoirs [6,52].

#### 7.1.3. Reservoir Management, Conformance Control, and Surveillance

- Successful projects rarely relied on viscosity alone to overcome channeling and heterogeneity. Tracers, pattern management, pressure surveillance, viscosity ramp-up strategies, injection allocation, conformance measures, and fracture management practices were frequently used to improve sweep efficiency and contacted reservoir volume [73,104,105].
- Implications for well pattern densification: Although the reviewed database does not provide sufficient information to establish universal well-spacing recommendations, several field observations offer guidance for future well pattern densification. Successful polymer floods increasingly relied on surveillance-driven reservoir management, including tracer studies, polymer breakthrough monitoring, injection pressure trends, and pattern-level production analysis to identify sweep inefficiencies and remaining oil targets. In mature projects, well pattern optimization was typically implemented as part of a broader reservoir management strategy rather than as a stand-alone intervention. The reviewed evidence suggests that densification may be most effective where surveillance indicates bypassed oil, weak sweep efficiency, incomplete pattern support, or significant heterogeneity between injectors and producers. Conversely, densification alone is unlikely to resolve performance limitations associated with channeling, fracture-dominated flow, injectivity constraints, or poor conformance. Therefore, future well pattern modifications should be guided by integrated surveillance evidence and reservoir connectivity assessments, ensuring that additional wells improve reservoir contact efficiency and polymer utilization rather than simply increasing development intensity.
- Integration of tracers with polymer injection improved diagnosis of sweep efficiency, reservoir connectivity, and polymer propagation behavior [102].
- Projects integrating pressure monitoring, injectivity trends, polymer concentration measurements, breakthrough behavior, viscosity monitoring, tracer data, and production surveillance were generally better able to distinguish reservoir response from operational or chemical problems and implement timely corrective actions [9,102].

#### 7.1.4. Operational Execution and Value Creation

- Early surface facility qualification minimized mechanical degradation before reservoir entry [99]. Reliable polymer preparation, hydration, filtration, oxygen control, and shear management consistently improved delivered polymer quality and viscosity preservation throughout the injection system [70,106].
- Decision speed has frequently proven to be as important as technical optimization. Several successful projects accelerated deployment by rapidly converting pilot observations into operational decisions and field implementation [68].
- Water-handling reduction emerged as a major value driver, improving project economics and, in some cases, reducing energy consumption and associated emissions [107]. While the magnitude of these benefits remained project-specific, they frequently strengthened project economics even under moderate oil price conditions.

### 7.2. What Consistently Failed

The database does not classify projects using a uniform binary success or failure criterion. The following observations should therefore be interpreted as recurring causes of underperformance, interruption, or failure to progress rather than calculated failure rates.

#### 7.2.1. Reservoir Conditions and Sweep Limitations

- Several underperforming projects involved reservoirs characterized by substantial water breakthrough, high heterogeneity, strong permeability contrasts, extensive channeling, fractures, thief zones, or unfavorable well connectivity prior to polymer flood startup [73,75,108]. Although delayed implementation may have contributed to reduced effectiveness in some cases, the available data do not allow separation of timing effects from the associated reservoir maturity and sweep efficiency challenges present at flood initiation.
- Unresolved channeling, poor conformance, and uneven sweep commonly resulted in early breakthrough, polymer recycling, poor pattern response, and reduced volumetric efficiency. These effects often limit the benefits achievable through viscosity increase alone.

#### 7.2.2. Injectivity and Design-Related Limitations

- Unsustainable injectivity and insufficient pressure margins represented recurring technical challenges. Projects encountered difficulties when polymer molecular weight, concentration, solution quality, injection rate, near-wellbore condition, or fracture behavior were incompatible with available pressure constraints [7,55,109].
- Aggressive viscosity or molecular weight targets frequently increased pressure gradients and injectivity risks without corresponding recovery benefits [9,110]. Field experience indicates that bulk rheology alone is insufficient as a design basis without adequate consideration of reservoir permeability, pressure limitations, fracture behavior, and injectivity constraints.

#### 7.2.3. Water Quality, Polymer Quality, and Surface Facilities

- Poor water quality control consistently contributed to degradation, plugging, filtration problems, unstable injectivity, and reduced polymer performance [94]. Oxygen, iron, suspended solids, bacteria, incompatible brines, incomplete hydration, and other contaminants were recurrent contributors to operational problems.
- Mechanical degradation in surface facilities reduced effective viscosity before reservoir entry [72]. Laboratory viscosities frequently overstated field performance when shear exposure, oxygen ingress, equipment configuration, or operational conditions were not adequately represented.
- Surface facility limitations, including inadequate polymer maturation, unsuitable pumps or valves, equipment downtime, tubing failures, storage constraints, and insufficient treatment capacity, reduced delivered polymer quality or interrupted injection [64,65,66,111]. These risks were often amplified in offshore, remote, or logistically constrained developments.

#### 7.2.4. Project Execution and Decision-Making

- Delayed response to early injectivity warnings, breakthrough anomalies, viscosity losses, or facility issues frequently increased the severity of operational problems [73,102].
- Misaligned pilot objectives and key performance indicators produced technically valid but commercially unconvincing outcomes [68]. Injectivity tests alone often failed to demonstrate incremental recovery, facility scalability, polymer utilization efficiency, or commercial viability.
- Advanced polymer chemistries expanded operating envelopes but did not eliminate limitations associated with reservoir heterogeneity, permeability, pressure capacity, water quality, facilities, logistics, or economics [22,55,68].

A limitation of the present review is that several potentially important project descriptors, including water cut at polymer flood initiation, reservoir depletion state, remaining oil saturation, injected polymer pore volume, and timing of polymer deployment relative to field life, were not reported consistently across the available field literature and therefore could not be incorporated into the quantitative analysis presented in Figure 5, Figure 6, Figure 8 and Figure 9 or Table 2 and Table 5. Consequently, the reported recovery and injectivity trends should not be interpreted as controlled assessments of deployment timing effects. Early polymer implementation has frequently been associated with improved outcomes in published field experience; however, timing is often coupled with reservoir heterogeneity, sweep efficiency, remaining mobile oil saturation, and water production history. Additional datasets containing consistent startup conditions would be required to separate these effects quantitatively [3,4,68].

### 7.3. Early Warning Indicators

The following indicators should be interpreted as operational diagnostics rather than definitive mechanistic evidence. No individual pressure, viscosity, breakthrough, or production observation is sufficient to diagnose performance independently.

#### 7.3.1. Pressure and Injectivity Behavior

- A sustained increase in injection pressure at constant injection rate warrants investigation, but it should not automatically be interpreted as injectivity impairment. During polymer flooding, pressure commonly increases as the polymer bank propagates, resistance factors develop, and the reservoir transitions from water injection to a more viscous displacement process. Interpretation therefore requires integration with injectivity index trends, polymer concentration, well tests, surveillance data, and operational history [7,38,73].
- Pressure increases may result from polymer bank propagation, resistance factor development, fracture growth or closure, near-wellbore effects, changing mobility conditions, or injectivity deterioration [56,71]. Conversely, unexpectedly low pressure or rarely high injectivity may indicate fracture extension, thief zone injection, loss of zonal containment, or preferential flow rather than favorable matrix injectivity [109].

#### 7.3.2. Polymer Breakthrough and Tracer Response

- Significant differences between predicted and observed polymer breakthrough timing may indicate uncertainty in transport property assumptions, reservoir connectivity, permeability architecture, heterogeneity, or sweep efficiency.
- Earlier-than-predicted polymer breakthrough commonly indicates stronger heterogeneity, preferential flow paths, fracture-assisted flow, or higher reservoir connectivity than assumed during project design [9,73,102]. Although retention, dispersion, and inaccessible pore volume may contribute to forecast uncertainty, unexpectedly early polymer arrival is more commonly associated with channeling through high-permeability zones or fractures.
- Tracer-confirmed channeling provides early evidence of reduced polymer utilization efficiency and poor sweep development [102]. Large differences between tracer and polymer arrival times may also provide insight into retention behavior, inaccessible flow pathways, fracture-assisted transport, or sweep redistribution and should trigger reassessment of reservoir models and flow path characterization [102,104].

#### 7.3.3. Polymer Quality and Surface Facility Performance

- Declining effective viscosity between the mixing facility and the wellhead may indicate mechanical degradation, oxygen exposure, incompatible water quality, biological activity, dilution effects, or equipment malfunction [9,72,94].
- Rapid viscosity loss between the mixing unit and wellhead is frequently associated with surface facility shear damage and warrants investigation of pumps, valves, mixing equipment, filtration systems, and oxygen control measures.
- Increasing filter ratio, differential pressure, stimulation frequency, or evidence of near-wellbore impairment may indicate deteriorating solution quality, suspended solids, incomplete hydration, bacterial contamination, or microgel formation rather than polymer adsorption alone [90,94].
- Repeated facility interruptions, unstable polymer concentration, inadequate maturation, dosing system failures, storage limitations, or off-specification injection may progressively reduce the effectiveness of the injected polymer bank even when nominal injection targets are achieved [70,111,112].

#### 7.3.4. Pattern Performance and Production Response

- Large differences in polymer arrival, water-cut response, oil rate response, or injection allocation among wells may indicate conformance problems, completion effects, reservoir heterogeneity, or poor voidage support and should be evaluated before field-wide design modifications are considered [75,108,113].
- Increasing polymer recycling, produced fluid handling difficulties, separation problems, fouling, filtration loading, or produced water reinjection constraints may indicate emerging facility bottlenecks before reservoir injection performance is affected [64,102,114].
- Interpretation should rely on integrated analysis of injection pressure and rate, injectivity index, polymer concentration and viscosity, water and solution quality, breakthrough behavior, tracer response, production performance, well test information, facility conditions, and reservoir history rather than any single observation alone [9,73,102].

### 7.4. Overall Integrated Field Lessons Discussion

Figure 12 condenses the database findings into three coupled requirements for successful polymer flood implementation: reservoir readiness and timing, polymer and injection system design, and quality control and surveillance. These pillars are interdependent rather than sequential. Reservoir architecture and remaining mobility control opportunity determine where polymer can be effectively propagated; polymer and facility design determine whether the intended solution can be delivered reliably; and surveillance verifies whether the expected mobility control and sweep efficiency improvements are being achieved under field conditions.

The first pillar, reservoir readiness and timing, emphasizes confirmation of a remaining mobility control opportunity, characterization of heterogeneity, connectivity, and fracture influence, definition of sustainable injectivity and pressure margins, and establishment of a pre-injection surveillance baseline. Field evidence demonstrates that reservoir architecture, flow path distribution, and sweep efficiency strongly influence project performance. Water-channeling or conformance-related issues were reported in 21 projects, highlighting the importance of understanding reservoir connectivity before polymer deployment [3,4,73]. Although earlier polymer implementation is frequently associated with favorable outcomes in published case histories, the reviewed database does not support deployment timing as an independent predictor of success because timing is typically coupled with reservoir maturity, remaining oil saturation, and heterogeneity effects.

The second pillar, polymer and injection system design, focuses on matching polymer chemistry and molecular weight distribution to reservoir conditions, prioritizing sustainable injectivity over maximum bulk viscosity, ensuring polymer stability under reservoir conditions, minimizing mechanical degradation during preparation and injection, and optimizing concentration, slug duration, and injection strategy. Injectivity- or pressure-related challenges were documented in 23 projects, making them the most frequently reported operational theme in the database. Polymer degradation or stability concerns were reported in 15 projects, while facility and logistics constraints were identified in 10 projects. Collectively, these observations indicate that scalable performance depends more on reliable polymer delivery and preserved in situ effectiveness than on achieving maximum laboratory viscosity [6,7,90].

The third pillar, quality control and surveillance, emphasizes maintaining polymer solution quality from preparation to injection, controlling oxygen, iron, solids, and bacterial contamination, and continuously monitoring pressure, injectivity index, viscosity, concentration, breakthrough behavior, and production response. Effective surveillance requires integrated interpretation of tracer data, polymer arrival trends, pressure behavior, and production performance to distinguish reservoir response from operational or chemical issues. Water quality or chemistry-related issues were documented in 14 projects. Recovery information was reported for 44 of the 54 project records, whereas polymer retention information was reported for only 23 projects, illustrating both the value of surveillance and the continuing limitations of public field reporting [9,70,102].

Across the reviewed evidence, polymer chemistry determines whether a formulation can be qualified for the intended reservoir environment, but chemistry alone does not determine scalability. Commercial progression consistently requires the simultaneous achievement of sustainable injectivity, effective reservoir contact, preserved polymer quality, reliable facilities, and surveillance-supported operational adjustment. Projects that underperform commonly exhibit weaknesses in one or more of these areas despite favorable laboratory results. Consequently, Figure 12 should be interpreted as an integrated field-derived decision framework rather than a prescriptive design recipe. Its purpose is to summarize what must be understood before injection, what must be delivered during implementation, and what must be continuously verified and adjusted throughout the life of a polymer flood [68,98].

## 8. Remaining Gaps and Future Research Needs

Despite substantial progress and a growing body of field evidence, several important challenges continue to influence polymer flood qualification, implementation, and scale-up. Future developments are likely to benefit more from improved prediction, monitoring, and decision support capabilities than from incremental polymer chemistry advances alone. Publicly available datasets remain incomplete, particularly regarding long-term injectivity evolution, polymer propagation, retention behavior, surveillance results, facility performance, and economic outcomes, limiting cross-project benchmarking and field-derived learning [9,68].

Polymer Science: A continuing research priority is improving the prediction of long-term polymer stability, propagation, and retention under representative field conditions. Many qualification workflows still rely heavily on short-duration laboratory testing that may not fully capture the combined effects of thermal exposure, oxygen ingress, mechanical degradation, water chemistry, and reservoir residence time. Future work should focus on establishing field-validated stability and propagation envelopes linked directly to documented project performance and long-term surveillance observations rather than laboratory metrics alone [21,68].

Reservoir Simulation: Although modern simulators reproduce the principal mechanisms of polymer flooding, uncertainty remains in representing injectivity evolution, retention dynamics, fracture-assisted flow, near-wellbore effects, and heterogeneous reservoir behavior. Future efforts should prioritize predictive frameworks that better integrate polymer transport, fracture behavior, surveillance information, and operational constraints to improve forecasting reliability and reduce uncertainty during project scale-up [1,115].

Surveillance and Digitalization: Field surveillance remains one of the least standardized aspects of polymer flood implementation. Greater integration of pressure, injectivity, polymer quality, tracer, breakthrough, and production data could improve recovery attribution, operational decision-making, and inter-project comparability. Future pilots should explicitly incorporate surveillance validation as a project objective and support broader application of digital workflows capable of integrating reservoir, operational, and chemical-performance information in real time [98,102].

Economics and Sustainability: Economic and sustainability assessments remain inconsistently reported across published polymer flood applications. Future evaluations should expand beyond incremental oil recovery to include polymer utilization efficiency, water-handling requirements, energy intensity, facility utilization, scale-up risk, and environmental performance metrics. More standardized reporting of economic and sustainability indicators would improve project benchmarking and support more robust investment decisions across different reservoir and operating environments [68,98,107].

While this review synthesizes lessons learned from a broad range of polymer flooding projects, opportunities remain to improve predictive design tools and benchmarking methodologies. Future work could focus on developing polymer flooding performance charts and screening frameworks for different reservoir classes by integrating reservoir quality, fluid properties, and operational parameters. In particular, parameters such as recoverable reserve ratio, permeability variation coefficient (Vk), mobility ratio (M), permeability contrast (Jk), oil viscosity, and flood maturity could be combined to establish performance envelopes and expected recovery ranges for different reservoir types. Such correlations may assist in project screening, uncertainty reduction, development planning, and optimization of polymer flooding deployment strategies.

## 9. Implications for Well Pattern Optimization and Densification

Field experience indicates that well pattern optimization should not be considered independently of the polymer flooding process being applied. In secondary polymer flooding projects, improved mobility control is established before extensive water channel development occurs, allowing conventional waterflood patterns to often achieve satisfactory sweep efficiency without significant modification. In contrast, tertiary polymer floods are frequently implemented in mature reservoirs where preferential flow paths and bypassed oil have already developed. Under these conditions, selective pattern densification, infill drilling, injector–producer realignment, or well conversion may improve sweep efficiency and enhance polymer utilization. Reservoir heterogeneity, oil viscosity, well spacing, and the timing of polymer deployment therefore collectively influence the degree to which well pattern optimization contributes to project performance. Across the reviewed case histories, successful projects generally match pattern design to reservoir architecture and flood maturity rather than applying a uniform spacing strategy, highlighting the importance of integrated reservoir management and polymer flood design workflows.

## 10. Conclusions

This paper consolidates published information and field-observed outcomes from more than two decades of polymer flooding into a comparative synthesis of technical, operational, and economic performance. The conclusions and implementation framework developed in this review are based on a synthesis of publicly available field literature and the compiled project database (with additional consulted material [108,109,110,111,112,113,114,115,116,117,118,119,120,121,122,123,124,125,126,127,128,129,130,131,132,133,134]). They should be interpreted as evidence-based engineering guidance rather than universally applicable rules. Conclusions are organized into six thematic groups:

### 10.1. Technology Maturity and Recovery Performance

- Polymer flooding has evolved into a mature and commercially deployed mobility control technology. Full-field developments such as Daqing, Pelican Lake, Mangala, Marmul, Captain, and Kalamkas demonstrate sustained injectivity, operational longevity, extensive surveillance, and expansion beyond pilot-scale applications.
- Reported incremental recoveries commonly range from approximately 8–20% OOIP, whereas values above 20% OOIP are less frequent and typically associated with favorable reservoir conditions, effective mobility control, sustained injectivity, and long project duration. Recovery performance is influenced by reservoir characteristics, deployment timing, project maturity, polymer dosage, and recovery baseline rather than by a single mobility criterion alone.

### 10.2. Reservoir Applicability and Screening Evolution

- Field experience confirms that polymer flooding can be successfully implemented across a broader operating envelope than traditionally assumed. Heavy oil projects, in particular, demonstrate that oil viscosity should be viewed as a mobility control and project-complexity variable rather than a strict screening limit.
- Temperature, salinity, hardness, permeability, and water quality are better interpreted as qualification variables than deterministic exclusion criteria. Advances in polymer chemistry have expanded opportunities in HSHT and carbonate environments, although commercial recovery validation remains more limited than evidence of injectivity and operational feasibility.

### 10.3. Polymer Systems and Recovery Mechanisms

- Advances in polymer chemistry have broadened potential applications but have not eliminated the importance of reservoir quality, heterogeneity, injectivity, connectivity, and operational execution. Polymer product selection should be regarded primarily as an implementation decision involving technical and economic trade-offs rather than as a distinct recovery mechanism.
- Available field evidence continues to support mobility control and sweep efficiency improvement as the dominant mechanisms responsible for recovery gains. Although laboratory investigations frequently report viscoelastic effects and residual-oil-saturation reduction, current field evidence remains insufficient to demonstrate a significant contribution to reservoir-scale recovery.

### 10.4. Injectivity, Retention, and Operational Execution

- Preserving injectivity remains one of the strongest determinants of project success. Performance depends on the combined effects of injectivity, reservoir quality, polymer propagation, mobility control, heterogeneity management, pressure control, and operational discipline rather than injected viscosity alone.
- Retention, degradation, and propagation remain important field-scale uncertainties. Consequently, field-validated data on polymer transport, retention, and viscosity stability provide greater practical value than isolated laboratory measurements. Successful projects consistently integrate water quality management, polymer quality control, pressure surveillance, tracer diagnostics, polymer monitoring, and timely corrective actions. The Matzen experience further demonstrates that polymer flooding can be sustained for more than a decade when these practices are maintained.

### 10.5. Economics and Implementation Strategy

- Economic performance is primarily controlled by incremental recovery, production acceleration, polymer utilization efficiency, injectivity sustainability, facility requirements, and reduced water-handling costs. While numerous projects report favorable economics and value creation, differences in recovery metrics, oil price assumptions, fiscal conditions, and reporting methodologies limit direct comparisons across fields.
- Polymer flood design strategies should be reservoir-specific. Differences in polymer concentration, slug size, tapered injection schemes, cyclic injection, and deployment timing primarily reflect variations in mobility control requirements, reservoir heterogeneity, injectivity constraints, economic objectives, and flood maturity. Successful projects align injection strategy with these reservoir and operational conditions rather than applying a single standardized design.
- Overall, field experience supports viewing polymer flooding as a risk-managed IOR process rather than a chemistry-driven tertiary EOR technology. Early qualification, staged scale-up, conservative injectivity management, surveillance-supported decision-making, and disciplined execution emerge as the principal drivers of success.

### 10.6. Implications for Future Polymer Flood Developments

- Recent growth in polymer flooding extends beyond the historically dominant regions of China, Canada, and the Middle East, with increasing activity in Latin America, India, offshore developments, and HSHT environments. This expansion reflects growing confidence in implementation practices, surveillance capabilities, and operational management rather than changes in the fundamental recovery mechanism.
- Future developments should recognize that:
  ○ Pilot results are not equivalent to mature commercial developments.
  ○ Project maturity influences performance analysis and recovery attribution.
  ○ Implementation quality is as important as reservoir screening.
  ○ Injectivity management remains critical throughout the project life cycle.
  ○ HSHT, carbonate, offshore, and heavy oil applications continue expanding beyond traditional applications, with many still in pilot or qualification stages.

### 10.7. Overall Key Learnings, Screening Guidance, and Future Outlook

The reviewed field evidence demonstrates that polymer flooding is a mature mobility control technology whose success depends more on reservoir qualification, injectivity preservation, surveillance, operational execution, and project management than on polymer chemistry alone. Reservoir temperature, salinity, permeability, oil viscosity, and water quality should be interpreted as qualification variables rather than strict exclusion criteria. Successful projects consistently employ staged qualification, pilot testing, surveillance-supported optimization, and disciplined scale-up. Future research should focus on improved prediction of polymer transport and injectivity behavior, field-scale performance benchmarking, development of reservoir-class-specific screening tools, and more consistent reporting of economic and sustainability metrics.

## Figures and Tables

**Figure 1:**
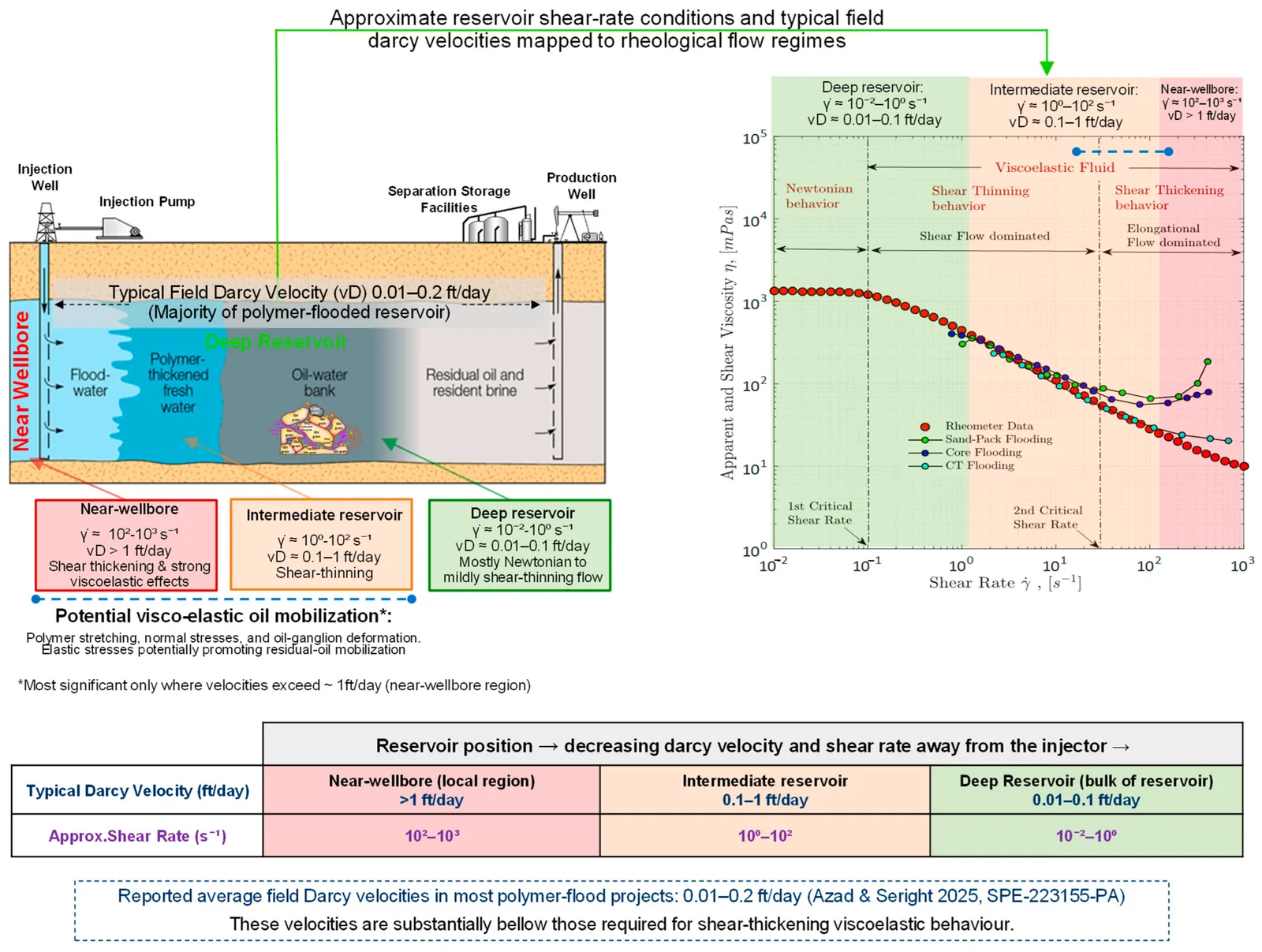
Conceptual relationship between reservoir position, Darcy velocity, apparent shear rate, and rheological flow regimes during polymer flooding. Fluid velocity and apparent shear rate generally decrease with distance from the injection well, resulting in a transition from near-wellbore conditions to bulk reservoir flow conditions. The figure illustrates potential rheological regimes, including near-Newtonian, shear-thinning, and localized shear-thickening or viscoelastic behavior [17,18]. Values and boundaries are schematic and intended for conceptual illustration only.

**Figure 2:**
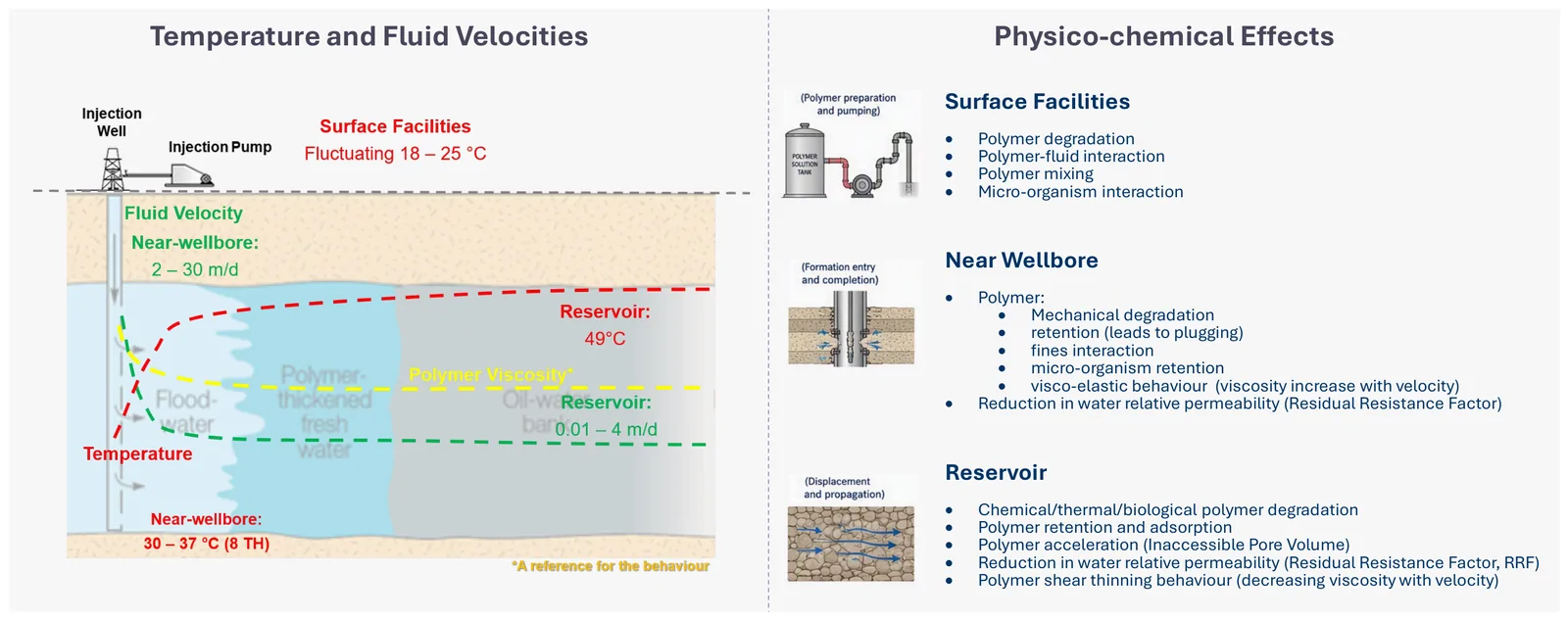
Conceptual representation of the principal conditions and physico-chemical processes affecting a polymer solution from surface preparation and injection through the near-wellbore region and into the reservoir. The temperature, flow velocity, and polymer viscosity profiles are qualitative and intended to illustrate potential trends rather than universal quantitative relationships. Actual behavior depends on facility configuration, completion design, injection rate, reservoir conditions, brine composition, polymer chemistry, rock properties, and degradation history. Localized mechanical degradation may occur in high-shear regions associated with wellbore flow, perforations, and near-wellbore flow convergence.

**Figure 3:**
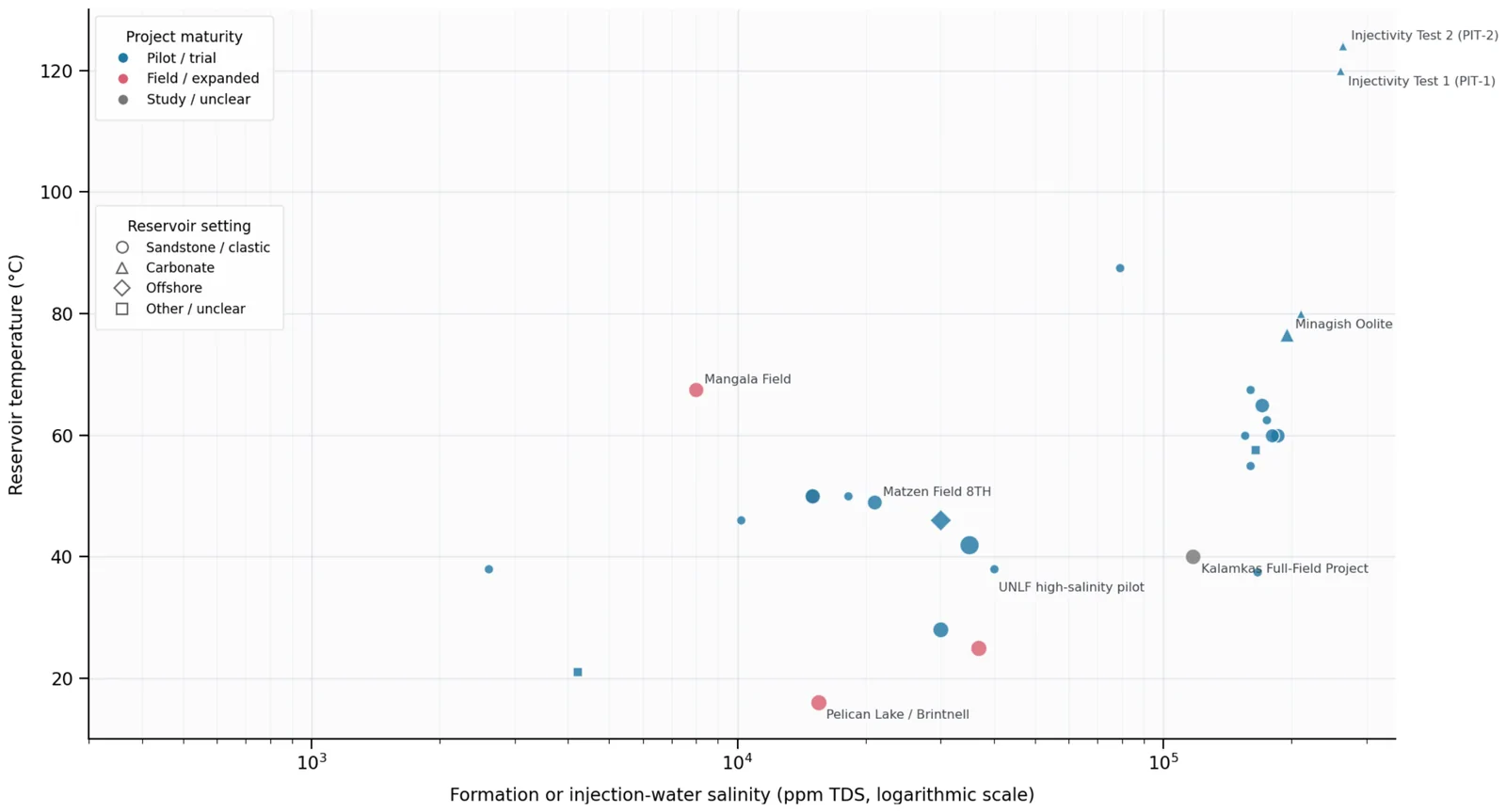
Documented operating envelope and project maturity for polymer flooding entries included in the review. Reservoir temperature is plotted against reported formation- or injection water salinity on a logarithmic scale. Color indicates project maturity, while symbol shape identifies sandstone/clastic, carbonate, offshore, and other or insufficiently classified reservoir settings. Bubble area represents reported percentage-based incremental recovery only where the recovery basis was sufficiently defined; fixed-size symbols represent projects for which recovery was not quantified or was reported on a noncomparable basis. Where temperature or salinity was reported as a numerical range, the midpoint was used solely for visualization. Of the 54 database entries, 31 contained sufficiently defined numerical values for both parameters and are shown. Projects lacking one or both numerical parameters were omitted. Because the recovery data differ in project scale, maturity, reporting basis, and observed or forecast status, bubble sizes are indicative and should not be used for direct performance ranking.

**Figure 4:**
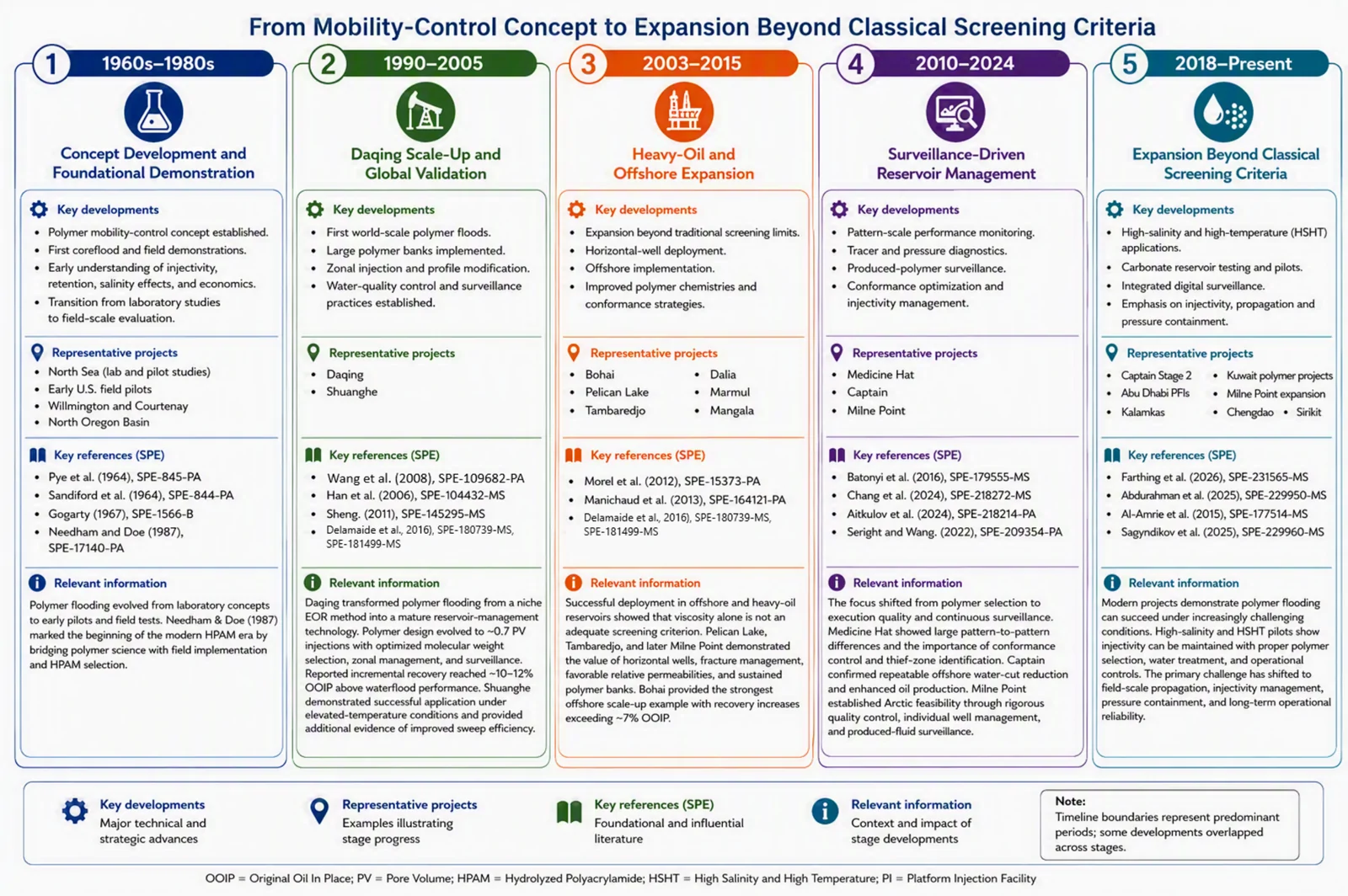
Evolution of polymer flooding from early mobility control applications toward deployment beyond traditional screening criteria. The timeline illustrates key stages in technology maturation, including large-scale field validation, heavy oil and offshore implementation, surveillance-driven reservoir management, and recent applications in high-temperature/high-salinity, carbonate, and other challenging reservoir environments [2,3,7,13,59,64,70,71,72,73,74,75,76,77,78,79,80,81,82,83].

**Figure 5:**
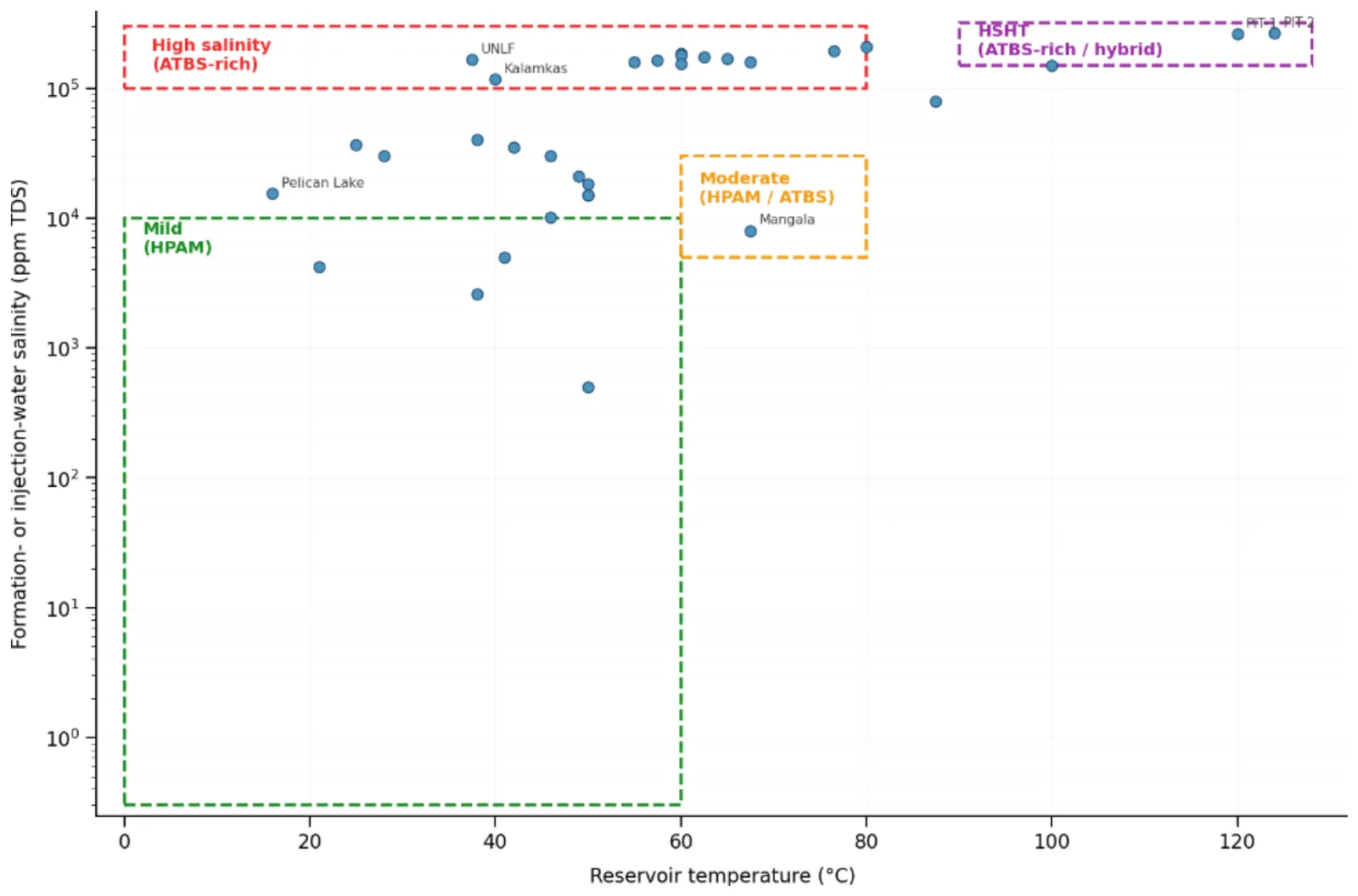
Reservoir severity cross plot for polymer flooding projects included in the reviewed database. Reservoir temperature is plotted against reported formation- or injection water salinity on a logarithmic scale. Dashed envelopes indicate the descriptive severity classes summarized in Table 2: Mild, Moderate, High Salinity, and high-salinity/high-temperature (HSHT). Of the 54 database entries, 32 contained sufficiently defined numerical values for both temperature and salinity and are shown. Where a parameter was reported as a numerical range, the midpoint was used solely for visualization; salinity values reported in g/L were converted to ppm TDS. The envelopes are indicative rather than universal screening boundaries, and the envelopes are indicative visualization ranges rather than universal screening limits. Reservoir conditions, project maturity, polymer system, recovery basis, and evidence quality differ substantially among entries.

**Figure 6:**
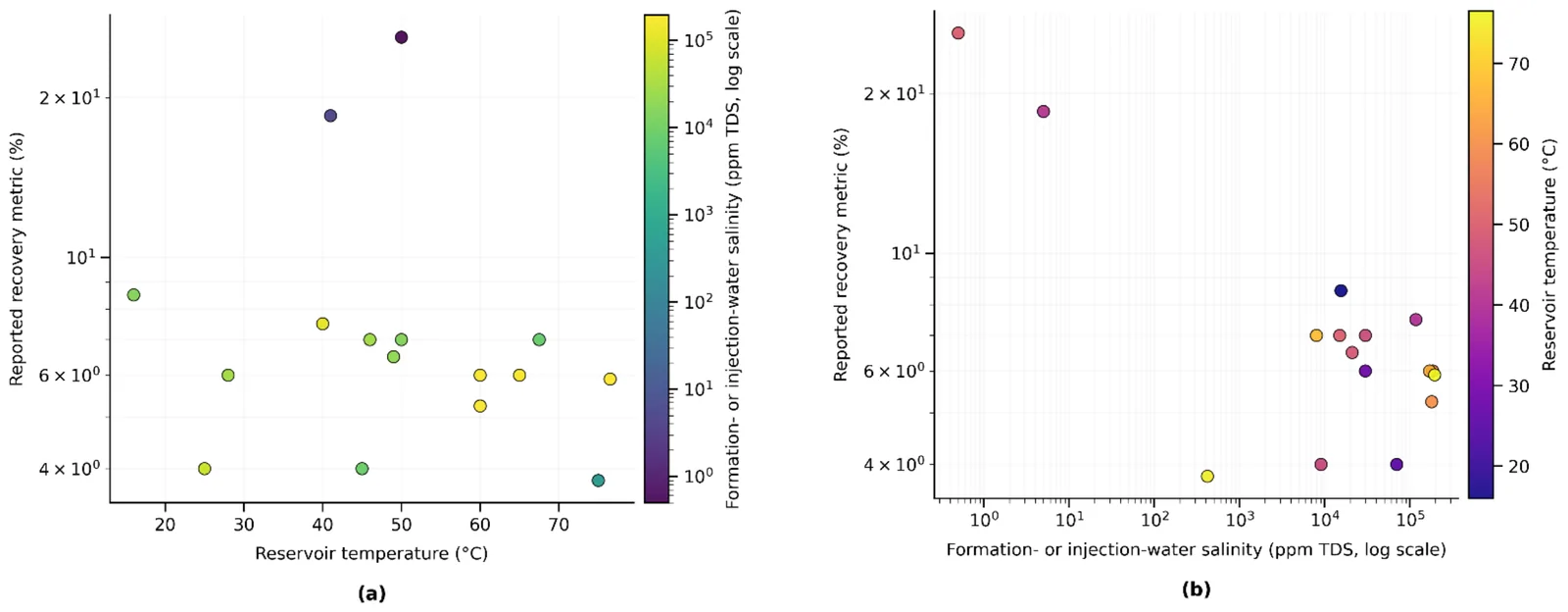
Reported recovery metrics for polymer flooding projects as a function of (**a**) reservoir temperature and (**b**) formation- or injection water salinity. Marker color represents formation- or injection water salinity in Panel (**a**) and reservoir temperature in Panel (**b**). Recovery is displayed on a logarithmic scale in both panels, while salinity is displayed on a logarithmic scale in Panel (**b**). The figure includes only projects reporting numerical recovery, temperature, and salinity values. Where source studies reported numerical ranges, midpoint values were used solely for graphical positioning. Recovery values retain their original reporting basis from the cited sources and may include incremental OOIP recovery, OIP-based recovery, recovery factor improvement, ultimate recovery factor, displacement efficiency estimates, or other percentage-based field performance indicators. Consequently, the figure is intended as a descriptive comparison of reported evidence rather than a direct quantitative ranking of polymer flood performance. The substantial recovery variability observed across comparable temperature and salinity conditions indicates that field performance is influenced by multiple interacting factors, including reservoir quality, oil viscosity, mobility ratio, heterogeneity, project maturity, injection strategy, injected polymer pore volume, and operational execution rather than temperature or salinity alone. Projects stating only injectivity performance, qualitative outcomes, absolute production volumes, or noncomparable recovery were excluded.

**Figure 7:**
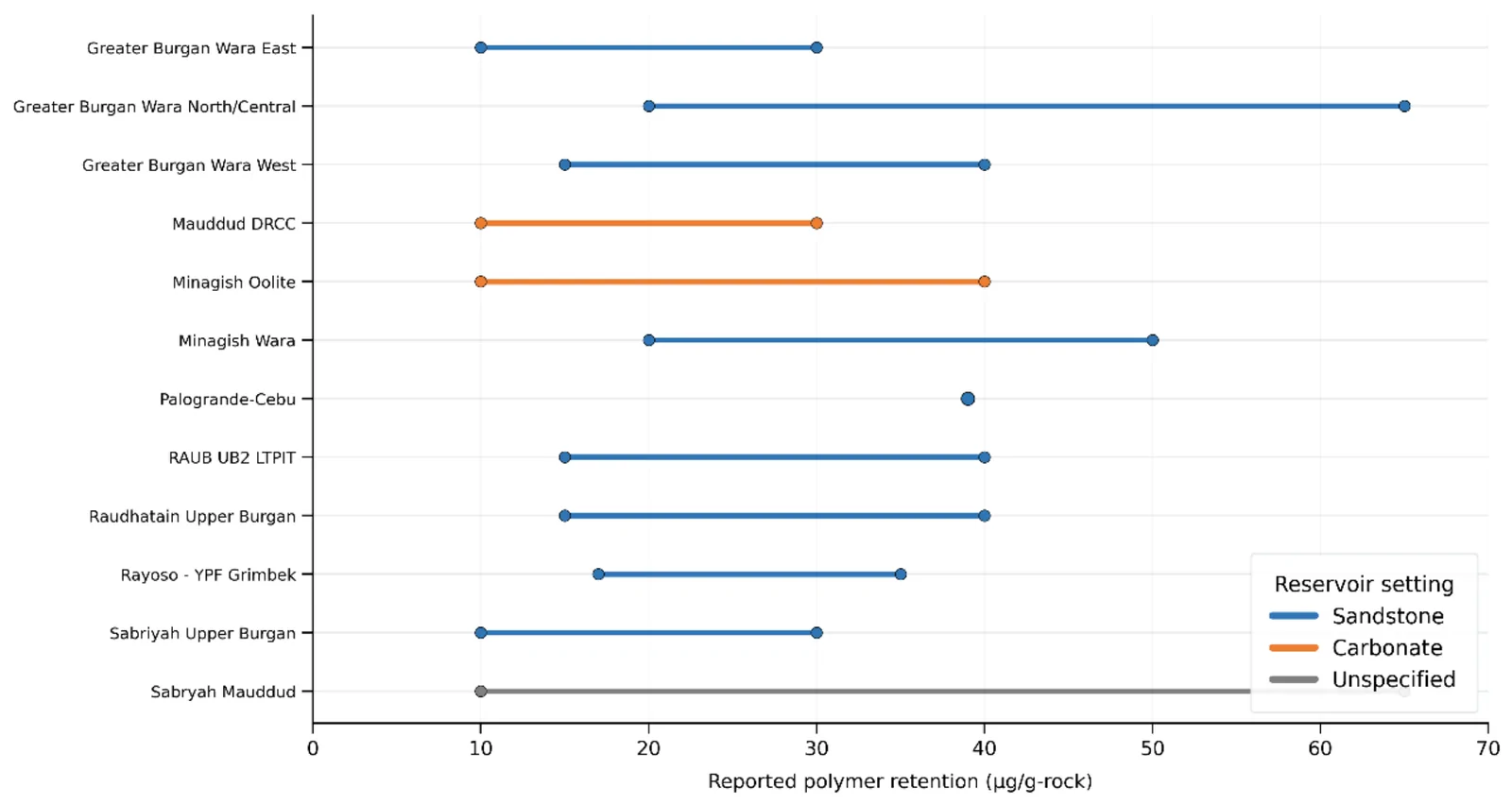
Reported polymer retention values for projects in the reviewed database that provide explicit numerical results in ug/g-rock. Horizontal lines indicate reported ranges for individual projects, while single markers represent point estimates. Colors denote reservoir setting. Of the 54 projects in the database, 23 report some retention information, but only 12 provide directly comparable numerical values in ug/g-rock and are therefore included. Qualitative descriptions, inferred values, percentage-based metrics, and non-comparable measurements were excluded. Reported values range from approximately 10 to 65 ug/g-rock and are predominantly from sandstone projects using HPAM or ATBS-modified polymers; only two carbonate projects provide explicit numerical data. The figure illustrates the extent and variability of the available project-level evidence and should not be interpreted as a statistical distribution, a universal retention range, or proof that retention systematically differs by lithology or polymer type. Comparisons should consider differences in mineralogy, permeability, brine composition, oil saturation, polymer formulation, experimental design, and analytical methodology.

**Figure 8:**
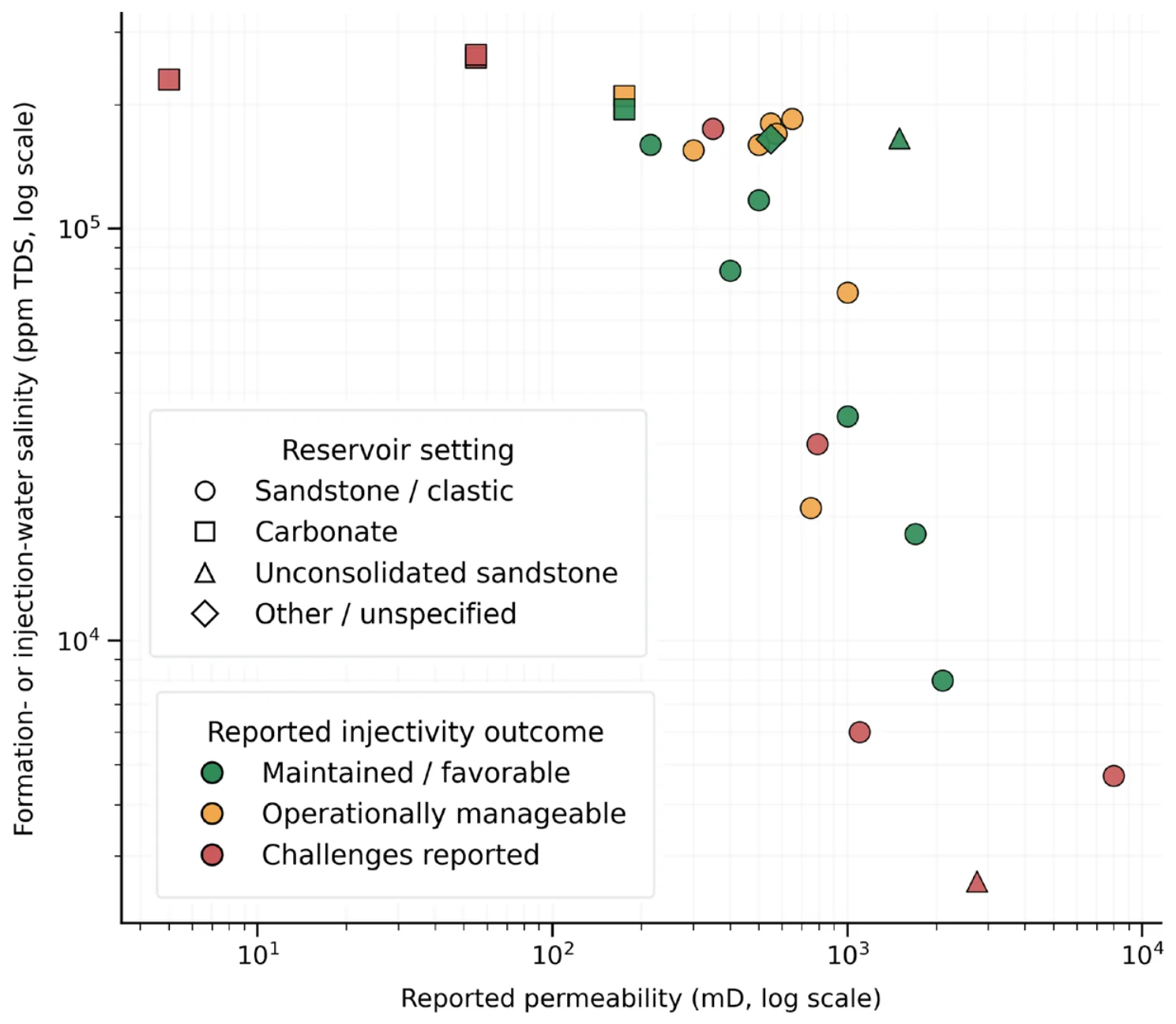
Reported injectivity outcomes for polymer flooding projects as a function of reservoir permeability and formation or injection water salinity. Marker color identifies the injectivity outcome reported in the reviewed source as maintained or favorable, operationally manageable, or challenges reported, while marker shape identifies the reservoir setting. The figure includes 25 entries from the 54-project database for which numerical permeability, numerical salinity, and explicit injectivity information were available. Where permeability or salinity was reported as a numerical range, the midpoint was used solely for graphical positioning. The injectivity categories summarize the project descriptions contained in the database and do not represent standardized injectivity index thresholds. The overlapping distribution of outcomes indicates that permeability and salinity alone do not determine injectivity performance. Polymer properties, molecular weight selection, water and solution quality, reservoir heterogeneity, completion design, fracture behavior, pressure constraints, and operational practices must also be considered. Projects lacking numerical permeability, numerical salinity, or an explicit injectivity assessment were excluded. The figure is intended as a descriptive synthesis of reported evidence rather than a predictive screening or risk-classification tool.

**Figure 9:**
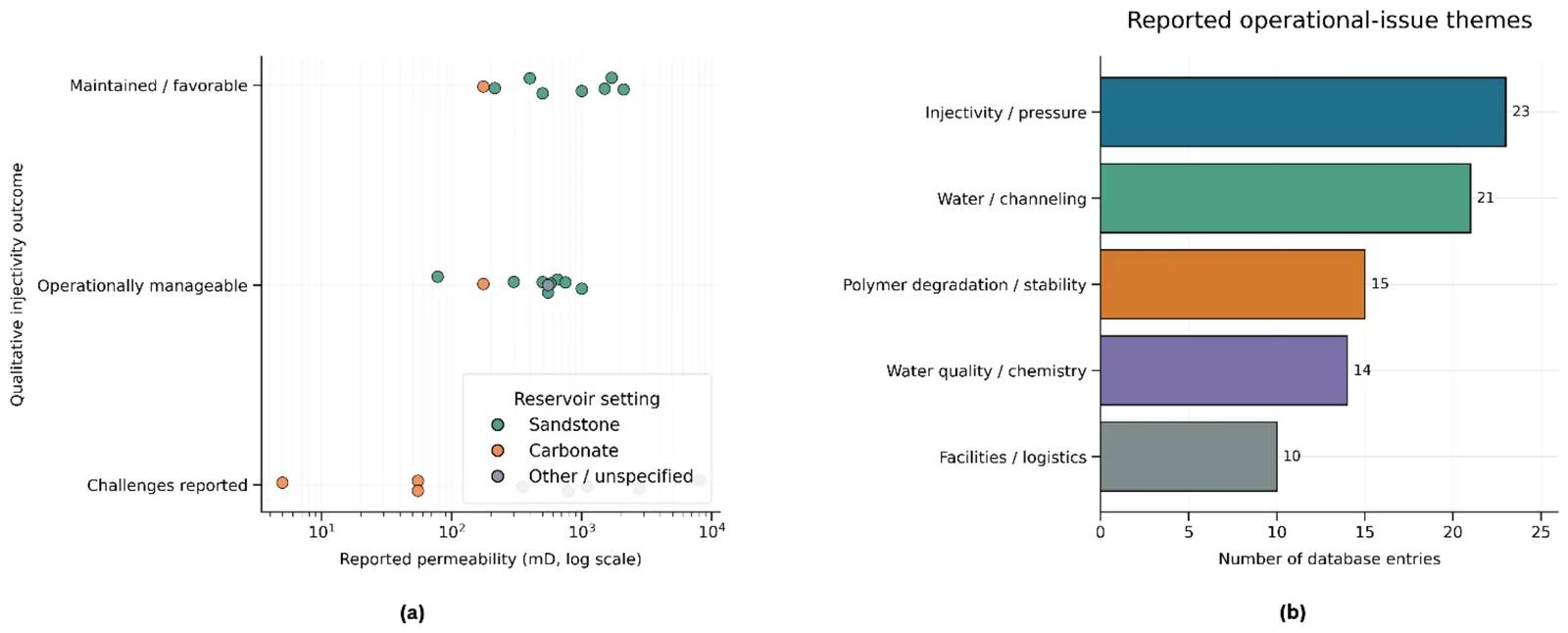
Injectivity responses and operational issue coverage in the reviewed polymer flood database. (**a**) Qualitative injectivity outcomes as a function of reported reservoir permeability for 26 entries containing both usable numerical permeability information and an explicit injectivity description. Where permeability was reported as a numerical range, the midpoint was used solely for graphical positioning. Injectivity outcomes were grouped as maintained or favorable, operationally manageable, or challenges reported according to the terminology and operational information recorded for each project. The categories do not represent standardized injectivity index thresholds. (**b**) Frequency of recurring operational issue themes across all 54 database entries. Injectivity or pressure was identified in 23 entries, water or channeling in 21, polymer degradation or stability in 15, water quality or chemistry in 14, and facilities or logistics in 10. Categories may overlap within individual projects. The figure provides a descriptive synthesis of reported evidence and should not be interpreted as demonstrating a deterministic relationship between permeability and injectivity performance.

**Figure 10:**
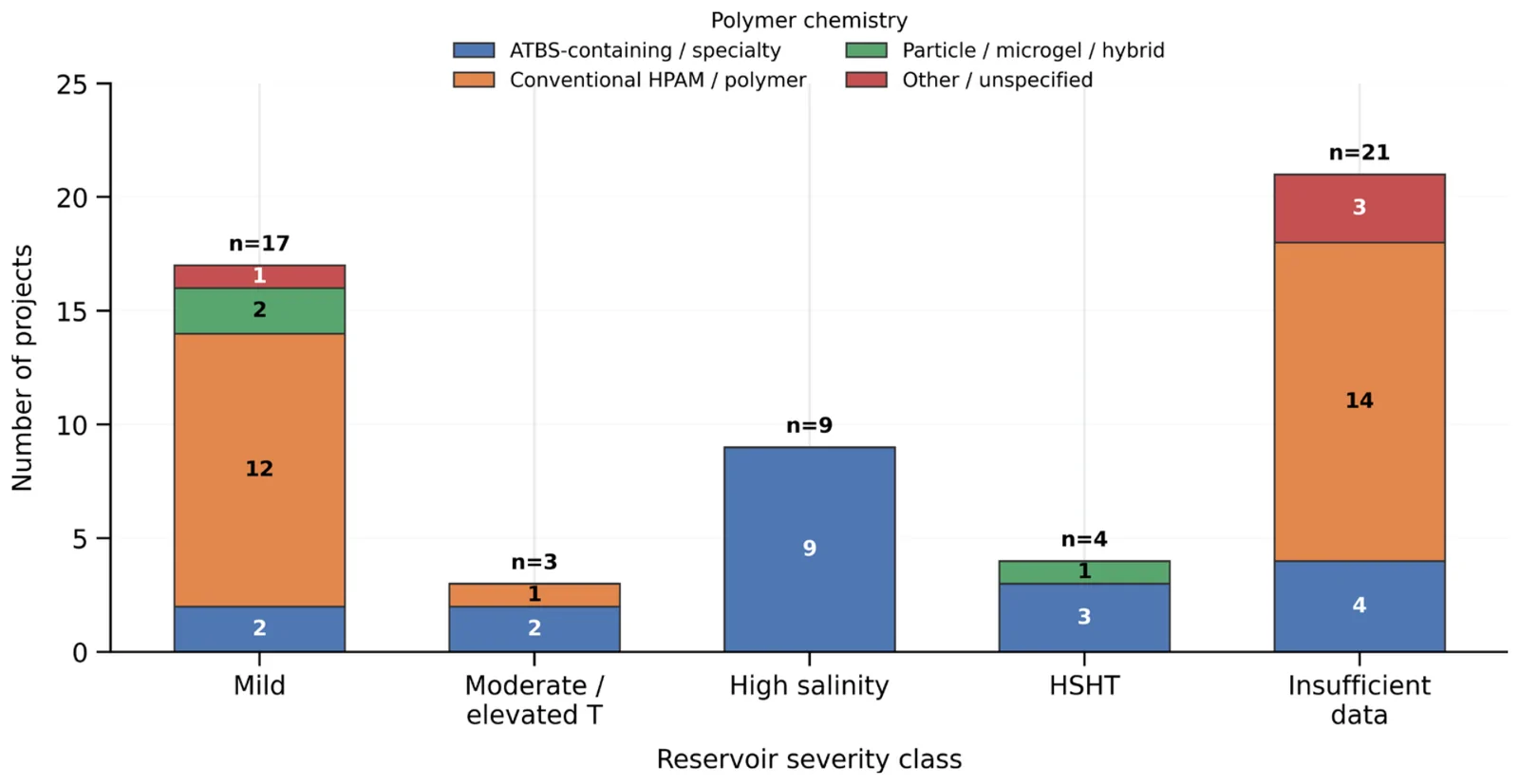
Polymer chemistry distribution by reservoir severity class for the 54 projects compiled in the updated database. Mild conditions correspond to salinity < 100,000 ppm and temperature < 60 °C; elevated-temperature conditions to salinity < 100,000 ppm and temperature ≥ 60 °C; high-salinity conditions to salinity ≥ 100,000 ppm and temperature < 80 °C; and HSHT conditions to salinity ≥ 100,000 ppm and temperature ≥ 80 °C. For reported ranges, midpoint values were used only for classification. Projects lacking sufficient salinity or temperature data are shown as “Insufficient data.” Stacked bars indicate project counts by polymer chemistry group. These classes are intended solely to visualize the compiled dataset and should not be interpreted as universal screening criteria, performance rankings, or evidence of the economic superiority of any polymer chemistry.

**Figure 11:**
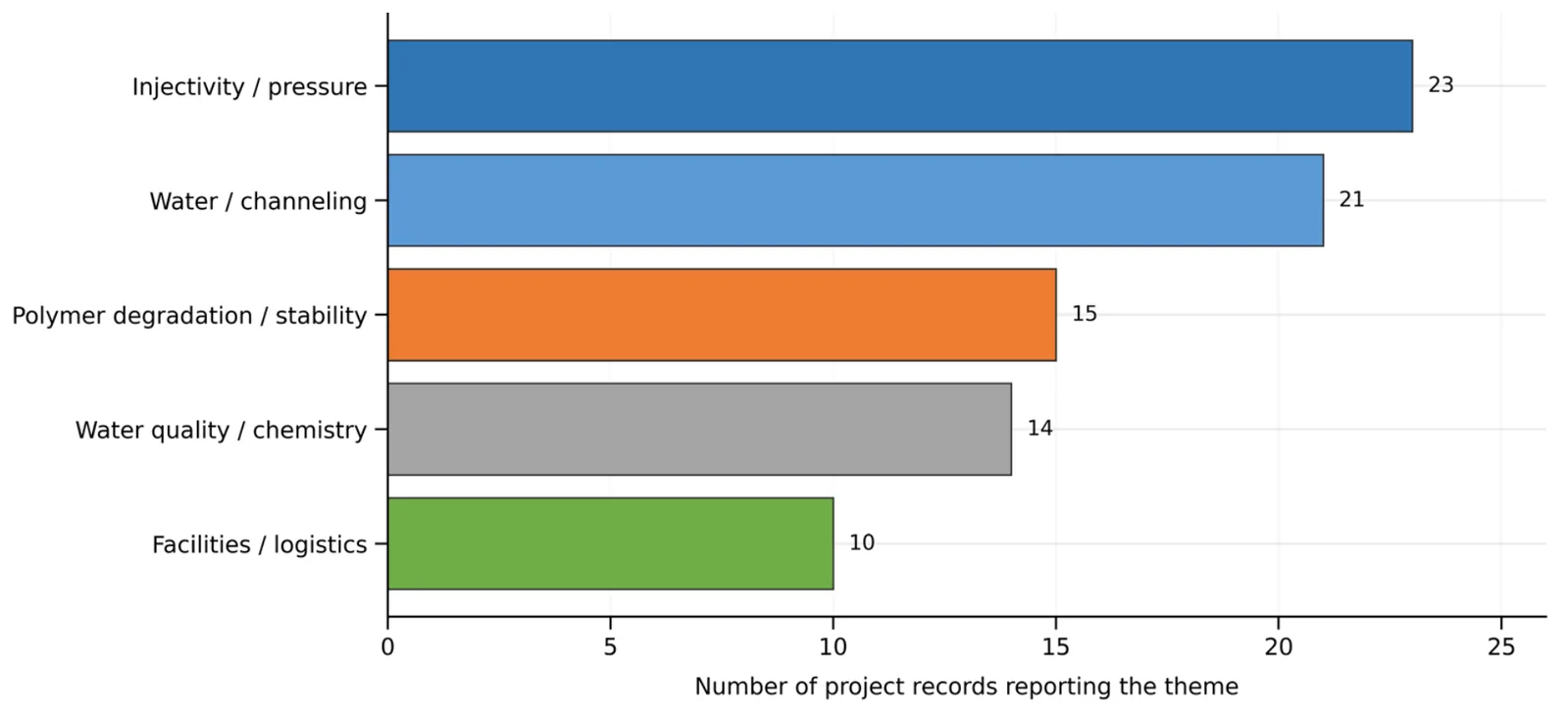
Recurring operational issue themes reported across the 54 polymer flood projects in the reviewed database. Individual projects may appear in multiple categories. Counts are based on qualitative coding of publicly reported project information and should be interpreted as indicators of reporting coverage and recurring implementation challenges, not as industry-wide failure rates, mutually exclusive failure mechanisms, or statistical risk rankings. Elevated injection pressure should not automatically be interpreted as injectivity impairment, as pressure increases may also reflect successful polymer bank propagation and mobility control development.

**Figure 12:**
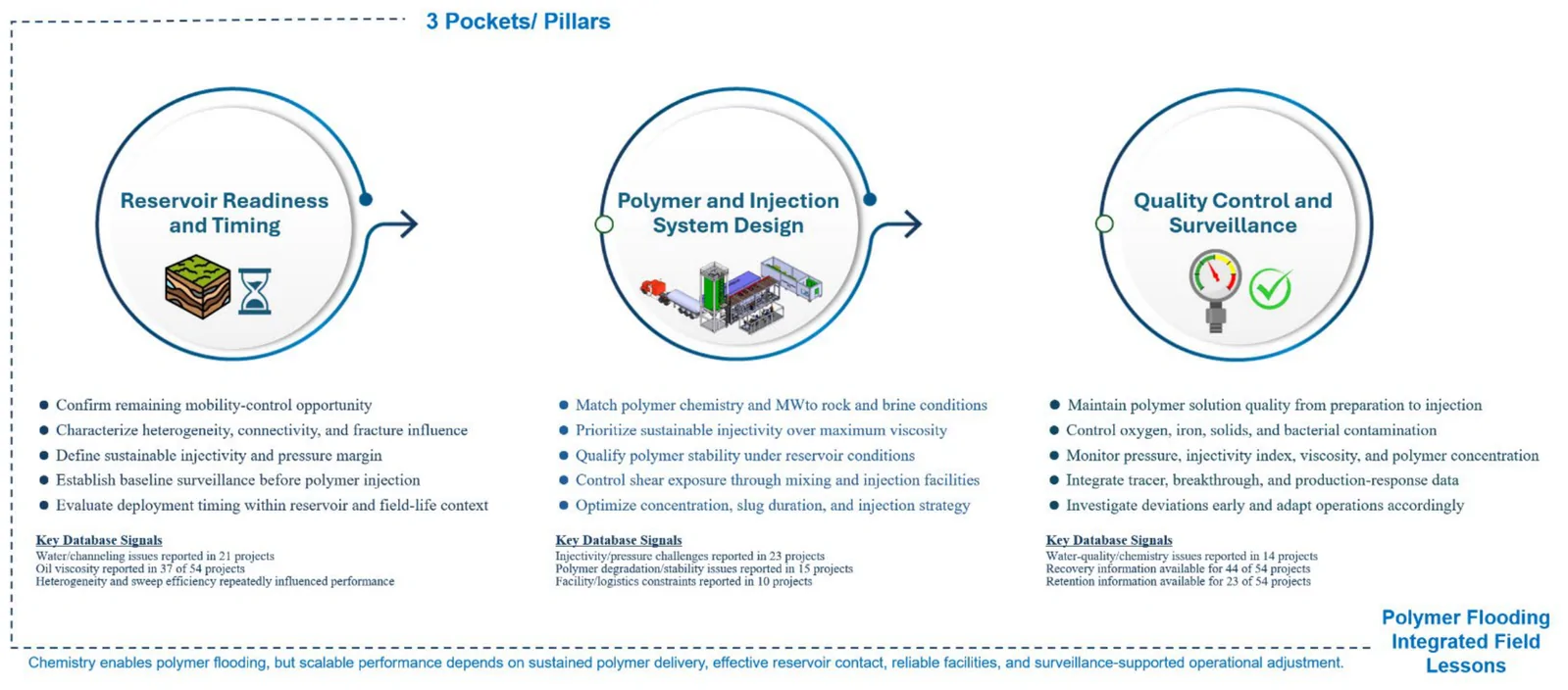
Integrated field lessons derived from 54 polymer flood project records. The framework organizes recurring field observations into three interdependent pillars: (1) reservoir readiness and timing, (2) polymer and injection system design, and (3) quality control and surveillance. Database signals highlight the most frequently reported operational themes, including water/channeling issues (21 projects), injectivity/pressure challenges (23 projects), polymer degradation or stability concerns (15 projects), water quality or chemistry issues (14 projects), and facility or logistics constraints (10 projects). Recovery information was reported for 44 of 54 projects, while retention information was reported for 23 of 54 projects. These categories may overlap and therefore represent reporting coverage rather than failure rates. Field evidence indicates that successful scale-up depends on the combined management of reservoir complexity, injectivity, polymer quality, facility reliability, and surveillance-supported operational decision-making rather than polymer chemistry alone [4,6,68,70,72].

**Table 1:** Overview of the polymer flooding dataset analyzed in this study. Counts represent project-level database entries and include field applications, pilots, injectivity trials, and supporting studies with publicly available technical information. Multiple reservoir intervals, implementation phases, or development stages reported as part of the same project are generally retained under one project entry. Parameter ranges and reporting counts are based only on entries containing sufficiently defined values; missing values were not estimated. The dataset spans projects initiated from 1985 to 2023, with several projects reported as ongoing or continuing beyond the latest documented reporting date. The numerical temperature envelope is 16–124 °C, and the documented salinity envelope used for comparison is approximately 500–270,000 ppm TDS.

Category	Sub-Category	Count/Range
Projects analyzed	Total project-level entries	54
Geographic coverage	Countries represented	20
Project start period	Earliest to latest reported start year	1985 ^1^–2023
Reservoir type	Sandstone/clastic	41
	Unconsolidated sandstone	5
	Carbonate	5
	Conglomerate/other	1
	Not sufficiently reported	2
Development maturity	Pilot/trial	39
	Full field/commercial	11
	Other/supporting study	3
	Unclear from available documentation	1
Operating environment	Offshore projects	8
	Onshore or not explicitly classified as offshore	46
Polymer chemistry	Entries explicitly identifying HPAM or an HPAM-based	37
	Other, unspecified, associative, particle-based, or hybrid	17
Temperature	Reported numerical range	16–124 °C
	Entries containing temperature information	46 of 54
Salinity	Comparative reported range	Approx. 500–270,000 ppm TDS
	Entries containing salinity information	44 of 54
Oil viscosity	Reported reservoir condition coverage	Approx. 0.95–80,000 mPa·s
	Entries containing oil viscosity information	37 of 54
Incremental recovery	Entries containing recovery-related information	44 of 54
Polymer retention	Entries containing retention-related information	23 of 54
Reference traceability	Entries containing reference short text	41 of 54

^1^ Well-known projects were included as references. Dataset limitations: Only publicly documented projects containing sufficient technical information for at least part of the comparative assessment were included. Missing values were not estimated. Reservoir categories reflect the primary lithological description available for each database entry and should not be interpreted as detailed sedimentological classifications. Project counts may differ among figures and appendix tables because each analysis includes only projects reporting the relevant parameter. Recovery information was not averaged because the database combines observed and forecast values, different recovery bases, absolute production volumes, displacement efficiency measurements, and qualitative outcomes. The inventory is a literature-based benchmarking dataset and not a complete global census of polymer flooding projects.

**Table 2:** Reservoir Severity Versus Polymer Flooding Evidence.

Severity Class	Temp. ^1^ (°C)	Salinity ^1^ (ppm TDS)	Polymer Systems Represented	Dominant Mobility and Implementation Context	Recovery Evidence in the Reviewed Database	Key Interpretation and Limitation
Mild	<60	<10,000	Predominantly HPAM; other mobility control systems in selected cases	Generally favorable conditions for polymer stability and viscosity generation. Performance nevertheless depends on oil viscosity, permeability, heterogeneity, injectivity, and well configuration.	Includes pilots and commercial developments with reported incremental recovery, but the recovery basis varies among observed, forecast, incremental, and ultimate values.	Mild temperature and salinity do not necessarily imply low project complexity, particularly in heavy oil, low-permeability, or highly heterogeneous reservoirs.
Moderate	60–80	Approx. 5000–30,000	HPAM and partially sulfonated or ATBS-modified HPAM	Applicable where polymer stability remains manageable but thermal exposure, brine composition, injectivity, and mobility contrast require more detailed qualification.	The database contains both field-scale applications and pilots, with reported outcomes expressed using nonuniform recovery definitions.	The class is transitional and should not be interpreted as a universal boundary between conventional and severe operating conditions.
High salinity	Generally, <80	>100,000	Primarily ATBS-rich or otherwise salinity-tolerant polymers; conventional HPAM in selected qualified cases	Chemical compatibility, viscosity retention, water treatment, molecular weight selection, and injectivity commonly govern feasibility.	Evidence is dominated by pilots, long-term injectivity tests, and selected field expansions. Several records report no directly comparable percentage-based recovery value.	Successful injection or propagation demonstrates technical feasibility but does not by itself demonstrate commercial-scale incremental recovery.
HSHT	>90	>150,000	ATBS-rich sulfonated polymers and selected hybrid systems	Qualification is commonly focused on long-term stability, water quality, pressure containment, retention, and matrix injectivity.	The reviewed entries are predominantly injectivity or technology qualification cases. Reported recovery values are limited, model-dependent, locally measured, or unavailable.	The evidence supports an expanded technical operating envelope, but not yet a broadly demonstrated full-field recovery performance range.

^1^ Indicative value. Note: Severity classes are descriptive groupings used to visualize the operating conditions represented in the reviewed database and should not be interpreted as universal screening limits. Temperature and salinity alone do not define polymer flood feasibility or water–oil mobility behavior. Project performance also depends on oil viscosity and mobility, permeability, heterogeneity, injectivity, polymer chemistry and molecular weight, water composition, well architecture, pressure constraints, and operational execution. Recovery outcomes were not averaged because the reported values differ in project maturity, evidence status, scale, and recovery basis. High-salinity and HSHT records are dominated by pilots, long-term injectivity tests, and technology qualification programs.

**Table 3:** Representative injectivity behavior and operational concerns reported.

Reservoir	Permeability Represented in Database	Reported Injectivity Behavior	Retention Evidence	Principal Reported Concerns
Higher-permeability sandstone and clastic reservoirs	Commonly >500 mD, with broad project-specific ranges	Frequently maintained or operationally manageable, although pressure increases and local injectivity losses are reported	Numerical values are available only for a subset, primarily between approximately 10 and 65 µg/g-rock	Channeling, fracture initiation or extension, pressure constraints, completion effects, polymer quality, and uneven flow distribution
Intermediate-permeability sandstone and clastic reservoirs	Approximately 200–500 mD in several reviewed cases	Polymer propagation has been demonstrated, but responses vary with molecular weight, concentration, permeability contrast, well design, and operating pressure	Limited and inconsistently reported	Near-wellbore pressure increase, skin, fracture activation, mechanical entrapment, and heterogeneity-driven redistribution
Low-permeability sandstone and clastic reservoirs	<200 mD in selected pilots	Both manageable injection and operational challenges are reported; reservoir-specific qualification is generally required	Sparse numerical coverage	Restricted pressure window, molecular weight selection, injectivity redistribution, stimulation requirements, and fracture-assisted flow
Carbonate reservoirs	Approximately 5–300 mD in the reviewed numerical cases	Greater uncertainty is reported; responses range from stable matrix injection to pronounced injectivity index decline	Only two explicitly classified carbonate entries provide numerical ranges, approximately 10–30 and 10–40 µg/g-rock	Resistance factor development, water quality sensitivity, fracture-controlled flow, heterogeneity, near-wellbore skin, and pressure limitations
Unconsolidated and heavy oil sandstone reservoirs	Commonly hundreds to several thousand mD	Injection may be maintained, but performance depends strongly on completion design, pressure limits, polymer quality, and fracture behavior	Generally unavailable or qualitative	Sand management, channeling, fracture extension, polymer recycling, water quality, and completion-related constraints

Note: The classifications summarize observations in the reviewed database and are not deterministic screening thresholds. Permeability alone does not establish polymer injectivity or propagation. The database contains explicit injectivity or pressure information for 23 of 54 entries, while numerical retention remains incomplete and should not be generalized by lithology. Reported permeability ranges were retained where possible and should be interpreted together with polymer molecular weight, concentration, water quality, reservoir heterogeneity, completion design, relative permeability, injection rate, and pressure relative to fracture initiation.

**Table 4:** Representative water quality considerations reported for conventional polymer and alkali-polymer (AP) flooding operations. Water quality requirements depend on polymer chemistry, reservoir conditions, source-water composition, produced water management strategy, and facility design. Conventional polymer flooding primarily seeks to preserve polymer viscosity and prevent mechanical, chemical, and biological degradation, whereas AP flooding imposes additional requirements related to alkali consumption, scale control, divalent ion management, and process stability. The AP-specific values reported in this table are largely derived from the Matzen AP water-softening field program and are presented as project-specific operational examples rather than universal specifications or screening criteria applicable to all polymer flood developments [94,95,96,97,99].

Parameter	Conventional Polymer Flooding	AP Flooding	Interpretation
Total dissolved solids	Project-specific. The reviewed database includes projects from low salinity to >250,000 ppm TDS using different polymer systems	Typically requires tighter conditioning according to alkali, polymer, and brine compatibility	Salinity alone does not define injectivity or polymer stability; polymer chemistry, divalent ion content, temperature, treatment, and residence time must be evaluated together
Ca^2+^ + Mg^2+^ hardness	Project-specific and managed according to polymer–brine compatibility and scaling risk	<5 mg/L after softening in the reported Matzen AP program	The Matzen value is a project-specific AP treatment target, not a universal conventional polymer flood specification
Dissolved oxygen	Minimized through facility design, oxygen exclusion, and scavenging where required	Same general requirement	Oxygen can accelerate oxidative degradation and viscosity loss
Dissolved iron	Managed through corrosion control, filtration, and water treatment	Same general requirement	Iron is associated with degradation and may contribute to solution quality or injectivity problems
Suspended solids and polymer impurities	Controlled through filtration, hydration quality, and polymer quality assurance	Same general requirement, with additional process-compatibility considerations	Solids, microgels, fisheyes, and high-molecular-weight contaminants may increase near-wellbore flow resistance
Oil in water	Controlled to limit fouling and filtration problems	≤30 ppm reported in the Matzen AP surface treatment operation	The numerical value is project-specific and should not be generalized
Back-produced polymer	Concentration is project-specific and may change after polymer breakthrough	Matzen pilot testing treated produced water containing approximately 400 ppm polymer	Produced polymer concentration is not governed by a universal operating limit
Apparent viscosity during water treatment	Project- and facility-specific	≤3 mPa·s reported for the Matzen treatment facilities	Surface treatment limits reflect facility hydraulics and operability rather than reservoir screening criteria
Water temperature	Project-specific	≥30 °C preferred in the reported Matzen treatment process	The Matzen value relates to surface treatment operability and is not a general reservoir-temperature requirement

**Table 5:** Economic Drivers, Reported Outcomes, and Operational Lessons from Polymer Flooding Field Applications. Reported economic and recovery values retain the bases used in the original field publications. Values should not be averaged or directly ranked without harmonizing recovery definitions, evidence status, cost boundaries, currencies, economic dates, oil price assumptions, and project maturity [1,7,84,87,98].

Economic Indicator/Driver	Updated Field Evidence	Relevance to Polymer Flooding	Interpretation and Limitations
Incremental recovery	Approximately 4% incremental OOIP at Baytex; approximately 7.5% field-wide incremental recovery at Kalamkas; 12–25% reported at Daqing; and 7–10% field recovery with ultimate estimates of 20–38% OOIP at Pelican Lake	Principal source of project value and production acceleration	The reported values use different recovery bases and include observed, estimated, and ultimate outcomes. They should not be averaged without standardization.
Polymer-related operating cost	Baytex reported polymer flooding OPEX at approximately 2× waterflooding OPEX, with polymer representing approximately 70% of incremental cost	Demonstrates the importance of polymer utilization efficiency	The reported cost contribution is project-specific and should not be generalized to all polymer floods.
Project value and profitability	Kalamkas: NPV of approximately USD 695 million and PI of approximately 1.6; Uzen: modeled PI of 1.93 and payback in Year 3; East-Messoyakhskoye: NPV of approximately RUB 125.4 million and PI of 1.40	Indicates potential commercial attractiveness when incremental recovery and operational performance are sustained	Results are based on different currencies, economic dates, assumptions, cost boundaries, and reporting scopes and are not directly comparable.
Polymer utility	Uzen: 50.6 t oil/t polymer; East-Messoyakhskoye: approximately 43 t oil/t polymer; Shuanghe: 0.7 bbl incremental oil/lb polymer	Relates polymer consumption to incremental production	Utility metrics require consistent attribution of incremental oil and standardized polymer consumption boundaries.
Unit-cost indicators	Courtenay/Châteaurenard: approximately USD 12/bbl total cost for 265,000 bbl of additional oil; Minagish Oolite: UTC of approximately USD 7–23/bbl; Medicine Hat: reported F&D cost of approximately USD 20/BOE	Provides project-level cost efficiency indicators	The metrics represent different cost definitions, development stages, and economic bases and should not be treated as equivalent.
Water-handling and efficiency benefits	Kalamkas: approximately USD 34.7 million in OPEX savings and around 60 MMbbl of water savings; Captain: lower water-handling requirements; Yariguí–Cantagallo: 6% improvement in energy intensity and carbon intensity reduction from 35.0 to 33.5 kg CO_2_/BO	May reduce lifting, treatment, disposal, and energy requirements	Financial benefits depend on water treatment capacity, disposal costs, facility ownership, energy demand, and baseline operating conditions.
Chemical volume optimization	Marmul slug optimization to approximately 0.5–0.7 PV was estimated to reduce polymer consumption by 26–36%	Reduces chemical expenditure while preserving the intended mobility control effect	The estimated savings are design-specific and depend on reservoir response, polymer concentration, and slug strategy.
Injectivity and operational reliability	Injectivity or pressure issues were identified in 23 of 54 records; water or channeling issues in 21; polymer degradation or stability issues in 15; and facility or logistics constraints in 10	Controls achievable injection rate, response timing, chemical delivery, and facility utilization	These are thematic record counts rather than failure rates because reporting detail and project maturity vary among the reviewed cases.
Principal economic sensitivities	Polymer cost and consumption, injectivity, well cost, facility CAPEX, water treatment, project scale, oil price, and timing of incremental response	Determine project robustness and investment attractiveness	The sensitivities should be assessed jointly because chemical selection alone does not determine project economics.
Preferred development strategy	Injectivity testing or pilot implementation followed by surveillance-informed, phased scale-up	Reduces technical and economic uncertainty before full-field investment	Improves confidence in injectivity, recovery attribution, polymer demand, facility design, and economic forecasts.
Economic-reporting limitation	Complete CAPEX, OPEX, cash flow, oil price, and polymer cost assumptions are unavailable for most reviewed projects	Prevents robust quantitative cross-project economic ranking	Economic examples should be assessed individually and retained on their originally reported bases.

**Table 6:** Illustrative influence of molecular weight distribution (MWD) on polymer transport and injectivity behavior reported in laboratory core-flood studies, Field-Flow Fractionation (FFF) measurements, and selected field observations. The table summarizes trends associated with dispersity and high-MW tail content that may influence injectivity, retention, propagation, and operational behavior beyond what is captured by bulk solution viscosity alone. Several relationships remain reservoir- and polymer-specific and should therefore be interpreted as qualitative observations rather than universal field outcomes [34,90,95].

Selection Criterion	Narrow MWD Polymer	Broad MWD Polymer
MWD characteristics	Lower dispersity and limited high-MW tail	Higher dispersity with greater presence of oversized fractions
Dissolution behavior	More rapid and uniform dissolution	Greater likelihood of dissolution heterogeneity when oversized fractions are present
Viscosifying efficiency	Predictable concentration–viscosity relationship	Viscosity may be disproportionately influenced by a limited ultra-high-MW fraction
Injectivity and permeability sensitivity	Generally lower injectivity impairment and reduced sensitivity to permeability restrictions	Greater likelihood of injectivity impairment where oversized fractions are filtered or retained in permeability-constrained media
Shear and extensional sensitivity	Lower extensional response generally reported under comparable conditions	Higher extensional response may occur when substantial high-MW fractions are present
Retention and resistance development	Retention may be more uniformly distributed; RRF commonly remains within reported ranges depending on test conditions	Preferential retention of oversized fractions and elevated RRF have been reported in some studies, subject to experimental conditions and retained polymer structure
MWD evolution during transport	Effluent MWD may remain closer to injected MWD	Progressive truncation of the high-MW tail and narrowing of the MWD may occur during porous media flow
Propagation and mobility control behavior	Earlier propagation and potentially more predictable mobility development	Delayed propagation may occur when oversized fractions are preferentially retained; mobility control performance may evolve as the retained fraction changes
Recommended application envelope	Often preferred where injectivity is a dominant design constraint	Requires additional qualification of injectivity, filtration, retention, and propagation behavior

## Data Availability

The database supporting this review was compiled from publicly available literature sources listed in references and summarized in Appendix A.1, Appendix A.2 and Appendix A.3. The dataset supporting this study is available from the corresponding author upon reasonable request.

## Abbreviations

The following abbreviations are used in this manuscript:

Abbreviation	Definition
ATBS	2-Acrylamido-2-Methylpropane Sulfonic Acid
EOR	Enhanced Oil Recovery
FFF	Field-Flow Fractionation
HPAM	Partially Hydrolyzed Polyacrylamide
HSHT	High Salinity High Temperature
IPV	Inaccessible Pore Volume
MWD	Molecular weight Distribution
NPV	Net Present Value
OOIP	Original Oil in Place
OPEX	Operating Expenditure
ppm	Parts Per Million
PV	Pore Volume
RF	Recovery Factor
RRF	Residual Resistance Factor
TDS	Total Dissolved Solids
µg/g	Micrograms per Gram
mD	Millidarcy
mPa·s	Millipascal Second

## Appendix A

### Appendix A.1. Summary of Main Information for Polymer Flooding Projects Used in This Study

**#**	**Project Name**	**Country**	**Region**	**Year Start**	**Year End**	**Reservoir Type**	**Oil Viscosity (mPas)**	**Ref.**
1	Aishwariya Lower Fatehgarh	India	Barmer Basin, Rajasthan	2017	Ongoing/full field	Lower Fatehgarh sandstone; moderately viscous oil	10–30	[106]
2	Alaska North Slope (Milne Point)	United States	Alaska North Slope, Milne Point Field	2018	2020	Unconsolidated sandstone	40–1300	[121]
3	Baytex 14-50-25W3	Canada	Saskatchewan (Lloydminster area)	2018	2022+	Lloydminster sandstone	≈2400	[14,124]
4	Bhagyam	India	Barmer Basin, Rajasthan	Pilot 2016; full field 2020	Ongoing	Multi-story fluvial sandstone; moderately viscous/bio-degraded zones	15–100; broader field descriptions up to 500	[116]
5	Bohai Bay Block-X/SZ36-1	China	Bohai Bay, offshore	2003	June 2019	Strongly heterogeneous heavy oil sandstone		[50,113]
6	Canto de Amarao	Brazil		2013		Sandstone	50–120	[118,119]
7	Captain EOR	United Kingdom	Outer Moray Firth, UK North Sea	2011 pilot	Ongoing; Stage 2	Offshore, highly permeable sandstone	40–140	[90]
8	Chengdao 22F	China	Shengli–Bohai offshore	2020	Ongoing	Offshore heterogeneous oil reservoir	30–70	[66]
9	Chichimene Fm1	Colombia	Llanos Basin, eastern Colombia	2015	At least end-2022	Deep, thick, high-temperature heavy oil sandstone	350–700	[130]
10	Courtenay/Châteaurenard	France	Paris Basin	1985–1989	November 1993	Elongated sandstone reservoir		[122]
11	Daqing (CNPC)	China	Daqing Oilfield	1990s	Ongoing	Sandstone	6–11	[29]
12	Diadema (CAPSA)	Argentina	—	2007	Ongoing	Sandstone	≈100	[111]
13	Diadema Pilot	Argentina	San Jorge Gulf Basin (Diadema Field)	2007	2014	Sandstone, highly stratified fluvial	≈100	[111]
14	East Bodo	Canada	Alberta side of East Bodo/Cosine	2006	NR	Upper Mannville “A”/sandstone	600–2000	[128]
15	East-Messoyakhskoye	Russia	Arctic Russia, Yamal-Nenets region	2017	2019	Unconsolidated Cenomanian sandstone (PK1-3)	≈111	[131]
16	El Corcobo Norte	Argentina		2012	2016+	Sandstone	160–500	[115]
17	EPIC001 Offshore Heavy oil	Brazil	Offshore Campos Basin (EPIC001)	2023	2024	sandstone	—	[119]
18	Greater Burgan Wara East	Kuwait	East Kuwait	2022		Sandstone	—	[56,57]
19	Greater Burgan Wara PF North	Kuwait	North/Central Kuwait	2022		Sandstone	—	[56,57]
20	Greater Burgan Wara West	Kuwait	West Kuwait	2022		Sandstone	—	[56,57]
21	HTHS Carbonate-Injectivity Test 1	United Arab Emirates	Onshore Abu Dhabi	2019	2019	Heterogeneous carbonate	—	[55]
22	HTHS Carbonate-Injectivity Test 2	United Arab Emirates	Onshore Abu Dhabi	2021	2021	Heterogeneous carbonate	—	[55]
23	JZ9-3 Polymer/SP Flood	China	Bohai Bay, offshore	2007	December 2014	Offshore sandstone	≈45	[117]
24	Kalamkas Full-Field	Kazakhstan	Western Kazakhstan, Mangystau	2014	2026+	sandstone reservoirs	≈23	[103]
25	Mangala Field	India	Rajasthan (Barmer Basin)	~2013	Ongoing	Sandstone (Fatehgarh)	8–22	[61,95]
26	Marmul	Oman	Southern Oman (Marmul Field)	1986	Present	Sandstone (medium-/heavy oil)	80–110	[52]
27	Matzen Field 8TH	Austria	Vienna Basin (Matzen Field)	2010	ongoing	Sandstone reservoir	16–19	[93,96]
28	Mauddud DRCC	Kuwait	East Kuwait	2023	Ongoing	Carbonate	—	[132]
29	Medicine Hat Glauconitic C (MHGC)	Canada	South-east Alberta (Medicine Hat)	2012	2018	Basal Quartz Sandstone	240–3000	[98]
30	Minagish Oolite	Kuwait	West Kuwait	2020	Ongoing	Oolitic carbonate	≈2.02	[56,57]
31	Minagish Wara	Kuwait	West Kuwait	2021	2023	Sandstone	—	[56,57]
32	Murphy Seal	Canada	Peace River Arch, Northern Alberta	2010	2016	unconsolidated sand	7000–15,000	[67]
33	NIM-R Polymer Injectivity Trial	Oman	South Oman	2014	2014	sandstone (clastic), horizontal well	250–800	[101]
34	Nimr-E Multiwell Trial	Oman	South Oman, Southern Central Salt Basin		cutoff 2022	Al-Khlata and Amin clastic reservoirs;	250–800	[100]
35	Dalia & Camelia (Angola)	Angola	Deep Offshore	2014	Ongoing	Sandstone	1–11	[64]
36	Offshore Abu Dhabi Carbonate	United Arab Emirates	Offshore Abu Dhabi, Arabian Gulf	2014	2014	Lower Cretaceous limestone carbonate	—	[109]
37	Palogrande-Cebu	Colombia	Upper Magdalena Valley Basin	2015	2017	Clastic sandstone	9–12	[83]
38	Patos-Marinza & Driza (Albania)	Albania	Onshore	2013	Ongoing	Sandstone	600–1600	[1,71]
39	Pelican Lake	Canada	Alberta	~2000s	Ongoing	Heavy oil sandstone	800–80,000	[71]
40	RAUB UB2 LTPIT	Kuwait	North Kuwait	2021	2022	Sandstone	—	[56,57]
41	Raudhatain Upper	Kuwait	North Kuwait	2021	2023	Sandstone	—	[56,57]
42	Rayoso-Grimbek	Argentina	Neuquén Basin	2016	Ongoing	Sandstone	≈120	[105]
43	Sabriyah Upper B.	Kuwait	North Kuwait	2019	2022	Sandstone	—	[56,57]
44	Sabryah Mauddud	Kuwait	North Kuwait	2022	ongoing		—	[56,57]
45	Shuanghe Elevated-Temperature Pilot	China	Henan Oilfield	1994	1996	Thick heterogeneous sandstone	≈70	[127]
46	Sirikit Oil Field	Thailand	Onshore Thailand	2019	2021	Fluviodeltaic sandstone	≈0.95	[104]
47	South Turgay Pilot	Kazakhstan	South Turgay Basin	2014	2015+	Multilayer, fractured, heterogeneous sandstone	≈1.68	[108]
48	SZ36-1 Pilot	China	Liaodong Bay, Bohai Bay, offshore	2006	NR	Offshore heavy oil sandstone, deltaic and heterogeneous	13–380	[114]
49	Tambaredjo-Sarah Maria	Suriname	Guiana Basin, onshore	2008	2012	Heavy oil sandstone reservoir (Tambaredjo T1/T2 sands)	1260–3057 cP (av. 1728 cP)	[84]
50	Umm Niqqa Lower Fars-High-Salinity 1-Spot Pilot	Kuwait	North Kuwait, Umm Niqqa Field (Lower Fars Reservoir)	2019	2019	unconsolidated sandstone	—	[82]
51	Uzen	Kazakhstan	Mangystau/Uzen Field	2022	2025	Jurassic sandstone, highly heterogeneous	≈4.5	[59]
52	XJ6	China	Xinjiang Oilfield (XJ6 Block)	2013	2015	Strongly heterogeneous multilayer conglomerate	—	[129]
53	Yariguí–Cantagallo TAP	Colombia	Middle Magdalena Valley Basin	2020	2023	clastic reservoir with thief zones and preferential flow channels	≈24	[91]
54	Zuata-Petrocedeño Pilot	Venezuela	Orinoco Oil Belt (Zuata Main Field)	2016	2018?	Unconsolidated Miocene sandstone	4000; dead-oil reports 9k–15k	[125]

### Appendix A.2. Summary of Key Technical Parameters for Projects in This Study

**#**	**Project Name**	**Permeability (mD)**	**Salinity (ppm)**	**Temp (°C)**	**Polymer Type**	**Pilot/Full Field**	**Incremental Recovery (%)**	**Polymer Retention (µg/g)**
1	Aishwariya Lower Fatehgarh	1000–5000	NR; relatively hard/divalent brine	Up to 77 at OWC	HPAM; ~25% initial hydrolysis	Injectivity test → six-well extension → full field		
2	Alaska North Slope (Milne Point)	500–5000	2600 (source water)	Very low (Arctic conditions)	HPAM	Pilot (two injector–producer patterns, horizontal wells)		
3	Baytex 14-50-25W3	1000	70,000 ppm TDS (3500 ppm hardness)	25	HPAM (Partially Hydrolyzed Polyacrylamide, MW > 20 MDa)	Secondary/Tertiary-Full Field (after waterflood)	~4% incremental OOIP (EUR increase from ~13% waterflood to ~17%)	—
4	Bhagyam	Multi-Darcy; exact value NR			NR in retrieved abstract	Pilot → full field		
5	Bohai Bay Block-X/SZ36-1	~2000 average	Field value not reported; Bohai laboratory injection water 9047 TDS	Field value not reported; representative Bohai tests at 65	Hydrophobically associating polyacrylamide, AP-P4	Single-well pilot → well-group trial → large-scale application	>7.1% OOIP as of June 2019	Not reported
6	Canto de Amarao	38–200	0.5	50	—	Tertiary	26	
7	Captain EOR	3000–11,000		31	Polymer; exact commercial grade NR in retrieved source	Pilot → Stage 1 → full-field Stage 2	NR; >15 MMbbl enhanced oil produced reported, not %OOIP	
8	Chengdao 22F				Powder-form polymer, 3000 ppm	Large-scale offshore, 43 injection wells	11.6 forecast, not observed	
9	Chichimene Fm1	100–10,000		“High temperature”; numeric NR		Five-pattern pilot	4–9% by pattern	
10	Courtenay/Châteaurenard		Fresh formation/injection water; numeric TDS not reported	30	Emulsion-form partially hydrolyzed polyacrylamide; 900–1000 ppm	Pilot → commercial field expansion	6.6% of pore volume, published basis; not stated as %OOIP	~35
11	Daqing (CNPC)	400–1000	3–7	41	HPAM (150–300 cP solutions)	Full Field	12–25	
12	Diadema (CAPSA)	10–5000	15,000	50	Flopaam 3630S (HPAM), 1500–3000 ppm	Tertiary	6–8% OIP	—
13	Diadema Pilot	High perm ~500 mD avg	15,000 ppm NaCl	50 °C	Flopaam 3630S (HPAM), 1500–3000 ppm	Pilot	6–8% OIP	N/A
14	East Bodo	NR in retrieved sources	Water chemistry variable; exact TDS NR		Associative polymer	Three-injector/six-producer pilot	20% OOIP laboratory forecast after waterflood; field % NR	
15	East-Messoyakhskoye	50–5000		16	HPAM polymer (≈1830 ppm; 30 cP at surface, 60–80 cP in reservoir)	Pilot	not clear	
16	El Corcobo Norte	500–4000	40,000	38	HPAM	Polymer injection pilot	-	
17	EPIC001 Offshore Heavy oil	N/A	Field-scale modeling values	N/A	HPAM (modeled), viscosity-optimized	Simulation study (pilot-scale)	Improved vs. waterflood (non-numerical)	Strong dependency on adsorption level
18	Greater Burgan Wara East	300–1000	100,000–270,000	50–70	ATBS-HPAM	Pilot	5–7% OOIP	10–30
19	Greater Burgan Wara PF North	300–800	120,000–240,000	50–70	ATBS-HPAM	Pilot	4.5–6% OOIP	20–65
20	Greater Burgan Wara West	250–900	100,000–240,000	50–80	ATBS-HPAM	Pilot	5–7% OOIP	15–40
21	HTHS Carbonate-Injectivity Test 1	Upper: 10–100+; Lower: 1–20	260,000	120	SAV10 (ATBS-rich sulfonated polymer)	Pilot (single-well injectivity test)		~50 (used in calibrated model)
22	HTHS Carbonate-Injectivity Test 2	Upper: 10–100; Lower: 1–20	264,000	124	SAV10 (ATBS-based, thermally stable polymer)	Pilot (single-well injectivity test)		~50 (model-selected mid-case)
23	JZ9-3 Polymer/SP Flood				Polyacrylamide; 0.12–0.15% 3640C in SP sequence	Three-year polymer flood followed by full-field SP	4% incremental oil from polymer phase	
24	Kalamkas Full-Field	>500 mD	~98,700–136,200 ppm TDS	40	HPAM (partially hydrolyzed polyacrylamide): Flopaam 5205 (5% ATBS) and Flopaam 1630S	Full-field expansion	~7.5% (field-wide average over waterflood)	retention ~20% lower vs. standard HPAM in lab/core tests
25	Mangala Field	200–4000	<8000 ppm	65–70	HPAM (+ ASP variant in later stages)	Polymer pilot → full-field	~7% improvement in RF	—
26	Marmul	N/A	High salinity (>150,000 ppm typical of Oman)	>100 °C (HT)	ATBS/HPAM (e.g., SAV10, 15 cP solutions)	Full Field	Improved recovery (exact % not given but significant incremental RF)	N/A
27	Matzen Field 8TH	500–1000	21,000	49	HPAM, 500–1000 ppm (20 MDa, 30% hydrolysis)	Pilot	Good incremental observed, 5–8%	not observed
28	Mauddud DRCC	50–300	150,000–270,000	70–90	Hybrid system (Zr/La crosslinker + Sulfonated HPAM)	Pilot	Not yet quantified	10–30
29	Medicine Hat Glauconitic C (MHGC)	580–1000+ mD	30,000 ppm	21 °C–35 °C	HPAM (Polyacrylamide), specifically FLOPAAM 3330	Pilot (5 horizontal injectors)	6% realized initially; targeted 10% with expansion	Inferred high due to low temperature and clay content (~10%)
30	Minagish Oolite	~50–300	150,000–240,000	70–83	ATBS-HPAM	Pilot	4.5–7.3% OOIP	10–40
31	Minagish Wara	200–800	110,000–210,000	45–65	ATBS-HPAM	Pilot (LTPIT)	Not reported (injectivity focus)	20–50
32	Murphy Seal	500–2000	Generally low to moderate TDS (Seal water is relatively fresh)	18–25	HPAM (Partially Hydrolyzed Polyacrylamide)	Initially a Pilot; expanded to wider field applications	Target ~10–15% over primary production/waterflood	Moderate; influenced by high clay content in some sand facies
33	NIM-R Polymer Injectivity Trial	200–2000 (avg. ≈ 800)	~6000 (reservoir brine)		HPAM polymers of low and high MW (including high-MW Flopaam 3630S)	Pilot (Long-term injectivity trial)	Not reported (injectivity focus)	
34	Nimr-E Multiwell Trial	NR in accessible text				Multiwell pilot: 3 horizontal injectors, 4 horizontal producers	~7% IOIP observed/assessed	
35	Dalia & Camelia (Angola)	1000	30,000	46	High-MW HPAM (hydrolyzed polyacrylamide)	Pilot	7	
36	Offshore Abu Dhabi Carbonate	5	230,000	Not explicitly stated	Thermally and calcium-tolerant polymers (two proprietary polymers, molecular weight optimized by controlled shearing) used with proprietary surfactant system	Pilot (single-well SP CEOR with SWTT)	Not reported as field RF; oil desaturation ΔSo ≈ 33–36% locally	
37	Palogrande-Cebu	6–150	—	62	HPAM polymers (FP3230, FP3330, FP5115; low–medium MW, partly sulfonated)	Pilot (two inverted patterns: PG-34, PG-37)	1.86% achieved (PG-34); projected up to ~8.8% (PG-34) and ~2.4% (PG-37)	~39
38	Patos-Marinza & Driza (Albania)	100–2500	high salinity	40–42	Flopaam 3630S (HPAM)	Field case	Polymer flooded areas reaching an ultimate recovery factor (URF) of 30%	—
39	Pelican Lake	3000+	1–30 g/L salinity	16	HPAM	Massive field PF	Recovery 7–10% OOIP (field), 26% (pilot), ultimate 20–38% OOIP	—
40	RAUB UB2 LTPIT	100–500	110,000–200,000	50–70	ATBS-HPAM (lower MW)	Pilot (LTPIT)	Not reported (injectivity focus)	~15–40
41	Raudhatain Upper	100–600	120,000–230,000	55–70	ATBS-HPAM (reduced MW)	Pilot (LTPIT)	Not reported (injectivity focus)	~15–40
42	Rayoso-Grimbek	200–1800	35,000	42	Flopaam 3630S (HPAM), 1500–3000 ppm	Pilots (modular/staged)	14%	17–35 µg/g
43	Sabriyah Upper B.	~30–400	120,000–200,000	~60–75	ATBS-HPAM (≈20–40% ATBS)	Pilot (LTPIT)	Not reported (injectivity focus)	~10–30
44	Sabryah Mauddud	~100–1000	89,000–241,000	~45–70	ATBS-HPAM (15–50% ATBS)	Pilot (LTPIT)	Not reported (injectivity focus)	10–65
45	Shuanghe Elevated-Temperature Pilot	420 average		75	Modified high-MW HPAM S525 and S625	Pilot	3.8 observed by December 1995; 9.8 forecast ultimate	
46	Sirikit Oil Field	35		85–95	HPAM polymer	Pilot	-	
47	South Turgay Pilot	~400 mD average (range 85–90, 2–2200 mD)	~79,000 ppm	85–90	HPAM terpolymer (acrylamide–acrylic acid–ATBS), SNF Flopaam	Pilot	Not reported (oil rate increased; water cut stabilized)	Not reported
48	SZ36-1 Pilot	NR field	High-salinity/high-hardness brine; exact TDS NR	NR	Hydrophobically associating polymer; AP-P4 identified in later work	Single-well pilot → broader application	Field % NR; laboratory associative-polymer displacement improvement 9.28%	
49	Tambaredjo-Sarah Maria	4000–12,000 field T1; laboratory packs 401–877	500 injection; ~4700 formation	38	Flopaam 3630S HPAM; 1000 → 1350 → 2300 ppm	Three-injector pilot; later expansion		Field typically 50–250; full calculated range 14–517; laboratory ~0 ± 20
50	Umm Niqqa Lower Fars-High-Salinity 1-Spot Pilot	1000–2000	166,000 ppm TDS	< 40 °C (≈ 35 °C reservoir)	ATBS-based HPAM	Pilot (one-spot SWCT-based)	≈ 54% additional displacement efficiency vs. produced waterflood	—
51	Uzen	1.3–120 mD	Cretaceous brine (preferred), lower salinity	62 °C	Flopaam 5205 VHM (5% ATBS), 1600 mg/L	Pilot → Field	Economic modeling: positive PI = 1.93 (incremental RF not numerically specified)	Lowest dynamic retention screened polymers
52	XJ6	Avg. 649 mD (up to ~1016 mD in S7^2^–S7^3^)	4212	21	Particle-type polymer system: SMG (Soft Microgel) with SLG + crosslinked polymer gel (LPG)	Pilot	—	—
53	Yariguí–Cantagallo TAP	Injector–producer ranges from ~400 mD up to 3000 mD (3 D)	Injection/formation water chloride ~17,500–18,800 ppm	35–65	Thermally Active Polymer (TAP): expandable sub-micron polymer particles (BWTR29404A, BWTR29398A) with surfactant dispersant	Pilot (two campaigns)	Not reported (%)-incremental oil reported as volumes (64,749 bbl total by December-2023)	NR
54	Zuata-Petrocedeño Pilot	15,000	10,200	46	HPAM (Flopaam 3630S)	Pilot	—	

### Appendix A.3. Summary of Project Performance or Projects in This Study

**#**	**Project Name**	**Injectivity**	**Viscosity Stability**	**Economic Notes**	**Operational Issues**
1	Aishwariya Lower Fatehgarh	Full-field ramp to 40–50 kB/D within one month; 25–30-cP wellhead viscosity	Serious produced-HPAM over-hydrolysis and precipitation documented	Shared Mangala polymer facility via ~14 km pipeline	ESP failures, strainer/PWRI fouling, pipeline pressure and increased pigging; calcium-carboxylate deposits
2	Alaska North Slope (Milne Point)	Short-term injectivity comparable to water; long-term injectivity declines as polymer fills reservoir	Target 45 cP maintained initially; effective viscosity reduced in situ by dilution with injected water	economic evaluation not yet reported	Declining injectivity over time; polymer slicing unit downtime; gas in source water; need to inject near fracture pressure
3	Baytex 14-50-25W3	Good injectivity; max WHIP ~5000 kPa reached at 50 cP	Very stable; minimal degradation, produced viscosity up to 95% of injected, typically stabilizes at 10–30%	Polymer flood OPEX ≈ 2× waterflood; polymer ≈ 70% of incremental cost; considered economic success	Channeling, high WOR spikes, max injection pressure limits; required gel conformance treatments
4	Bhagyam	Field challenges reported, but deployment achieved		Full-field deployment selected after successful pilot	Early water breakthrough; injectivity, conformance, surface and model-history-match challenges
5	Bohai Bay Block-X/SZ36-1	Average injection pressure rose 7.2 → 8.9 MPa, ~24%	Long-term field propagation demonstrated; associative polymer selected because conventional HPAM weakened under high salinity/hardness	~4.8 million m^3^ incremental oil from 99,183 t cumulative polymer; unit economics not reported	Platform-space limits; pressure rise; polymer/reservoir compatibility; scaling and produced water reinjection risks [ythx.scu.edu.cn]
6	Canto de Amarao				
7	Captain EOR	Repeated field responses; six new subsea injectors in Stage 2	Offshore design includes polymer handling and non-shearing equipment	Enabled otherwise negative-NPV infill C64; improved oil rate and reduced water handling	Polymer manufacture, shipping/storage, back-produced polymer management, topside/subsea complexity
8	Chengdao 22F	~32,000 B/D; 90–130 barg WHP; >98% injection system reliability	Non-shearing choke valves and shortened maturation used to preserve viscosity	Powder selected despite higher CAPEX to reduce transport and OPEX; ~12 t/d polymer	Footprint, logistics, maturation, mechanical degradation and produced water treatment
9	Chichimene Fm1	Repressurization and WOR control observed	Numeric viscosity retention NR	Lower energy/carbon intensity evaluated against baseline	350–700-cP oil, broad permeability range, large thickness and pattern-scale variability
10	Courtenay/Châteaurenard	~380 m^3^/d during commercial injection	Injected viscosity ~21 cP; produced polymer characterization found no degradation	Additional 265,000 bbl reported at ~$12/bbl total cost; operation described as profitable	Produced polymer lagged simulation; pressure support and oil bank attribution required careful interpretation
11	Daqing (CNPC)	Very good; sustained over decades	Stable due to moderate T and salinity	Largest polymer flood globally	None major; well-developed operations
12	Diadema (CAPSA)	—	—	—	—
13	Diadema Pilot	Good; maintained even at 3000 ppm	Stable; viscosity ↑ where Fe^2+^ low	Strong oil rate gain; water cut ↓ 97.5% → 83%	Water quality critical; channeling; injection unbalance
14	East Bodo	Increased injection pressure and breakthrough observed	Target viscosity difficult to maintain because of water quality	Pilot intended to test commercial fieldwide reserve capture	Water quality, viscosity target, polymer breakthrough, bottom water and gas cap
15	East-Messoyakhskoye	Good; stable injection without near-wellbore plugging up to ~78 atm	Stable under Arctic conditions; no significant mechanical or chemical degradation observed	NPV ≈ 125.4 million RUB; PI = 1.40; ~43 t oil per t polymer	High water quality requirements; uncertainty in fracture pressure; optimal viscosity for full-field not yet defined
16	El Corcobo Norte	Injectivity loss reported, and pressure limit of 4700 kPa was achieved	achieved with no major operational issue		No major problems with Polymer plant operation and water–oil separation
17	EPIC001 Offshore Heavy oil	Injectivity loss increases with shear-rate modeling	Viscosity improves recovery but increases computational cost	Better economics at low adsorption; high adsorption kills NPV	Adsorption and shear impact injectivity and economics
18	Greater Burgan Wara East	Good	Stable (≈5 cP)	Positive NPV	None critical
19	Greater Burgan Wara PF North	Good	Stable (≈3.6–5 cP)	Economically favorable	Injectivity fluctuations managed
20	Greater Burgan Wara West	Good	Stable (≈7 cP)	Strong economics	RRF increased locally
21	HTHS Carbonate-Injectivity Test 1	Injectivity index declined from ~8 to ~0.8 B/D/psi; no fracture contribution	Stable under HTHS; additional 10–20% in situ viscosity loss inferred	Economics not evaluated; technology derisking phase	Initial skin buildup linked to water quality; shear-thickening risk at high rates; injectivity decline with polymer bank growth
22	HTHS Carbonate-Injectivity Test 2	Injectivity index declined from ~10 to ~0.9 B/D/psi; stable injection without fractures	Stable; viscosity control within ±10% of target; no shear-thickening observed	Economics not evaluated; supports future SIMGAP/SIWAP pilots	| Improved water quality mitigated injectivity impairment; no polymer-induced damage
23	JZ9-3 Polymer/SP Flood	8 injectors/23 producers in full-field SP stage		Oil rate doubling and water-cut reductions reported in responsive wells	Offshore chemical logistics; only 11 of 23 producers showed obvious response
24	Kalamkas Full-Field	No injectivity loss in pilots; injectivity comparable to waterflood; fractures aid injectivity	High when using PSU units; minimal degradation with oxygen control; severe viscosity loss observed with eductor units	NPV ≈ USD 695 million; PI ≈ 1.6; OPEX savings ≈ USD 34.7 million from reduced water handling; water savings ≈ 60 MMbbl	Large mobility contrast challenges. High salinity and iron in brine; oxygen-induced polymer degradation; eductor units caused up to ~45% viscosity loss; need strict QA/QC and nitrogen blanketing
25	Mangala Field	Good injectivity; horizontal well design	Stable under field conditions	Strong economics; major Indian PF success story. World’s largest polymer flood	Water cut management; need polymer quality control
26	Marmul	Good; sustained long-term injectivity achieved	Stable under high T and salinity; SAV10 formulation specifically engineered for HTHS conditions	Polynomial slug optimization: 0.5–0.7 PV saves 26–36% polymer	None major; long-term reservoir temperature requires advanced polymer stabilization
27	Matzen Field 8TH	Good injectivity. In some cases, injectivity improved by biocide treatment (~15% gain)	Polymer degradation ~20% in situ (stability moderate)		Past activities reported polymer degradation and microbial activity before biocide
28	Mauddud DRCC	Good	High thermal and chemical stability	Advanced DRCC concept	Zr/La chemistry control
29	Medicine Hat Glauconitic C (MHGC)	Challenging; required pressure monitoring due to shallow depth	Maintained 44–60 cP; sensitive to high shear and bacterial activity	F&D cost ~$20/BOE; aimed to reduce operational life by 60+ years	Rapid breakthrough (48 hrs), “thief” zones, and recycling of polymer
30	Minagish Oolite	Excellent (≤6000 bpd)	Stable (up to 7.5 cP)	UTC ≈ $7–23/bbl	Tight fracture pressure window
31	Minagish Wara	Good	Stable (≈5 cP @ 1500 ppm)	Scalable PF candidate	Minor injectivity loss. Fracture-assisted flow interpretation
32	Murphy Seal	High initially			Performance drops significantly in zones where oil viscosity exceeds 14,000 cp
33	NIM-R Polymer Injectivity Trial	Matrix injectivity achievable at 150–250 m^3^/d; fracture initiation occurred at higher rates/viscosities	Polymer rheology from lab adequately explained in situ behavior; stable viscosities of 50–100 cP achieved during injection	Trial aimed to de-risk full-field polymer flooding and potential polymer savings via low-salinity hybrid (no economics quantified)	Unexpectedly low fracture pressure (≈7100–8300 kPa) led to fracture initiation/re-opening; changing well conformance and fracture propagation dominated injectivity response
34	Nimr-E Multiwell Trial	Pilot injectivity problems were mitigated	GRE-lined tubing used to limit degradation	Polymer selected over thermal alternatives based on economics, energy efficiency, CO_2_ intensity and complexity	Water coning, polymer loss to aquifer, produced polymer impact on reed beds, zonal-conformance control
35	Dalia & Camelia (Angola)		Polymer maintained viscosity at high salinity	5+ million bbl polymer injected by 2011; first-of-its-kind offshore EOR	Logistics challenges: deep-offshore, FPSO integration, large well spacing, high salinity
36	Offshore Abu Dhabi Carbonate	Injectivity limited; polymer injectivity not demonstrated at ~5 mD (injectivity index dropped from 0.44 to 0.06 m^3^/d/bar)	Polymers designed for HT/HS stability; mechanical degradation applied; stability proven at lab scale but field injectivity constrained	Objective was technical proof-of-concept rather than economics	Polymer injectivity insufficient in very low-k zone (~5 mD); injectivity lower than design expectation; need for multi-well pilot
37	Palogrande-Cebu	Slight reduction; injectivity broadly similar to waterflood, manageable with WHP ≤ 2400 psi	Moderate degradation observed in backflow test (~40% viscosity loss); affected by oxygen/iron control	Pilot technically successful	Administrative interruptions, polymer degradation risk (oxygen/iron), injectivity redistribution, well reactivations required
38	Patos-Marinza & Driza (Albania)	Good injectivity in heavy oil, but reduction in injectivity	—	—	—
39	Pelican Lake	Excellent injectivity in horizontal well patterns	Very stable at low T and modest salinity	Economically one of the largest PF projects	Sand issues; VRR complexities
40	RAUB UB2 LTPIT	Improved after design change	Stable post-optimization	Demonstrated scale-up viability	Early MW selection issue
41	Raudhatain Upper	Moderate → improved	Polymer MW too high → injectivity decline. Stable after polymer switch	Viable after optimization	Early injectivity failure with high-MW polymer
42	Rayoso-Grimbek	Good injectivity; retention acceptable	Stable; negligible degradation reported	Modular pilots reduced CAPEX, Strong incremental response	Water cut remains high; adsorption higher at salinity
43	Sabriyah Upper B.	Excellent (≤2000 bpd)	Stable (4.6–4.8 cP at 1600 ppm)	Commercially viable rates achieved	Permeability reduction (RRF ≈ 2.4)
44	Sabryah Mauddud	Excellent (matrix flow)	Stable at 1200–1600 ppm (3–5 cP in situ)	Positive UTC; feeder for commercial design	Near-wellbore skin required acid stimulation
45	Shuanghe Elevated-Temperature Pilot	High-MW polymer successfully injected	Thermal-degradation prevention specifically required	Reported utility 0.7 bbl incremental oil/lb polymer	Thermal stability, heterogeneity and need for profile modification
46	Sirikit Oil Field	Initially no injectivity issue; deterioration observed in several injectors over time, mitigated operationally	Polymer viscosity partly degraded during injection (designed ~7 cP; interpreted in situ ~3 cP from fall-off test)	Estimated positive net cash flow to date; final economic evaluation pending due to ongoing pilot and operational constraints	Injectivity deterioration; need for mechanical degradation (ROs); tubing leakage and ESP blockages; voidage replacement control challenges
47	South Turgay Pilot	Adequate injectivity. Injectivity stable; bottomhole pressure and injection rates varied insignificantly	Stable at reservoir conditions	Positive economics indicated by reduced water production and stabilized oil rate	Presence of fractures and high-permeability channels; need for viscosity ramp-up to control channeling
48	SZ36-1 Pilot	Significant response after eight months; injectivity/formation damage requires surveillance	Associative chemistry selected because conventional HPAM weakened in saline/hard brine	Demonstrated offshore feasibility where fresh water and platform space were limited	Limited platform footprint; no freshwater source; high hardness; laboratory evidence of adsorption/retention and possible core damage
49	Tambaredjo-Sarah Maria	Polymer injectivity poor without fractures; hydraulic fractures (~20 ft) increased injectivity dramatically. Observed polymer injectivity 0.40–0.63 (B/D)/psi.	Excellent. Improved anaerobic sampling showed polymer propagated >300 ft with essentially no loss of viscosifying ability. Produced polymer retained 31–34 cP at 896 ppm and 1600 ppm TDS.	Not reported. Paper discusses improved sweep and positive oil-response but provides no economic metrics.	Initial injectivity insufficient; injection above formation-parting pressure required. Short hydraulic fractures were created. Channeling stronger in some patterns (e.g., 1N062-1N11 trend). Produced polymer measurements were highly sensitive to oxygen contamination during sampling.
50	Umm Niqqa Lower Fars-High-Salinity 1-Spot Pilot	Excellent; polymer injected at ~100–120 bbl/d with sub-ambient wellhead pressure	Stable during pilot; polymer maintained ~30 cP (±20%) at 10 s^−1^ with oxygen control and additives	Use of produced water eliminates need for desalinated or low-salinity water; expected cost and logistics benefits	H_2_S presence required oxygen removal and scavengers; strict filtration and QC required; short test period and wellbore volume effects
51	Uzen	Good after MW optimization; commissioning ramp-up defined	Stable to reservoir temperature and salinity	Payback Year 3; polymer utility factor 50.6 t oil/t polymer	Water quality constraints (dissolved O_2_ <200 ppb, solids, iron); produced water unsuitable
52	XJ6	Injectivity maintained with moderate pressure increase (~2 MPa); water injection and liquid production increased ~20%	Stable during pilot; effective mobility control despite low temperature and heterogeneity	Economic data not reported; pilot demonstrated strong production response and sweep improvement.	Well and pump failures caused shutdown of 4 producers during injection phase; extreme heterogeneity and strong channeling required complex slug design
53	Yariguí–Cantagallo TAP	No injectivity loss; injection rate maintained (~600 bbl/d); fracture pressure not exceeded (≈3300 psi)	Stable after activation; viscosity increases after thermal activation and stabilizes after ~4 days	Reduced water handling cost; 6% improvement in energy intensity; carbon intensity reduced from 35.0 to 33.5 kg CO_2_/BO	Need for strict water-rate control; one event of overdosing due to flow fluctuations; requires careful QA/QC and single-well injection control
54	Zuata-Petrocedeño Pilot	Sustained; mobility decreased from ~342 to ~145 mD/cP over time	Good stability before injection; viscosity reduced by mixing with reservoir brine	Feasibility-oriented pilot; economics not quantified	Mobility reduction over time due to polymer retention, salinity mixing, possible shear degradation
